# Respiratory syncytial virus–approved mAb Palivizumab as ligand for anti-idiotype nanobody-based synthetic cytokine receptors

**DOI:** 10.1016/j.jbc.2023.105270

**Published:** 2023-09-19

**Authors:** Julia Ettich, Christoph Wittich, Jens M. Moll, Kristina Behnke, Doreen M. Floss, Jens Reiners, Andreas Christmann, Philipp A. Lang, Sander H.J. Smits, Harald Kolmar, Jürgen Scheller

**Affiliations:** 1 Institute of Biochemistry and Molecular Biology II, Medical Faculty, Heinrich-Heine-University, Düsseldorf, Germany; 2 PROvendis GmbH, Muelheim an der Ruhr, Germany; 3 Institute of Biochemistry, Heinrich Heine University Düsseldorf, Düsseldorf, Germany; 4 Institute for Organic Chemistry and Biochemistry, Technical University of Darmstadt, Darmstadt, Germany; 5 Institute of Molecular Medicine II, Medical Faculty, Heinrich-Heine-University, Düsseldorf, Germany; 6 Center for Structural Studies, Heinrich-Heine-Universität Düsseldorf, Düsseldorf, Germany; 7 Centre of Synthetic Biology, Technical University of Darmstadt, Darmstadt, Germany

**Keywords:** synthetic cytokine, Palivizumab, interleukin 6, Fas, apoptosis, scFv, gp130

## Abstract

Synthetic cytokine receptors can modulate cellular functions based on an artificial ligand to avoid off-target and/or unspecific effects. However, ligands that can modulate receptor activity so far have not been used clinically because of unknown toxicity and immunity against the ligands. Here, we developed a fully synthetic cytokine/cytokine receptor pair based on the antigen-binding domain of the respiratory syncytial virus–approved mAb Palivizumab as a synthetic cytokine and a set of anti-idiotype nanobodies (AIP^VHH^) as synthetic receptors. Importantly, Palivizumab is neither cross-reactive with human proteins nor immunogenic. For the synthetic receptors, AIP^VHH^ were fused to the activating interleukin-6 cytokine receptor gp130 and the apoptosis-inducing receptor Fas. We found that the synthetic cytokine receptor AIP^VHH^gp130 was efficiently activated by dimeric Palivizumab single-chain variable fragments. In summary, we created an *in vitro* nonimmunogenic full-synthetic cytokine/cytokine receptor pair as a proof of concept for future *in vivo* therapeutic strategies utilizing nonphysiological targets during immunotherapy.

Cytokine receptors are in a monomeric off-mode and execute signal transduction in a dimeric or multimeric on-mode after cytokine binding (1). The on-mode can be interrupted and converted to the off-mode by depletion of the cytokine from the cytokine receptor, natural cytokine antagonists, or intracellular negative feedback mechanisms. Among others, antibodies as synthetic cytokine antagonists and agonists are a recent but promising development representing an own class of future therapeutic biomolecules (2, 3). Moreover, the approved chimeric antigen receptor (CAR) T-cell therapy for severe cases of acute lymphatic leukemia (4) is based on synthetic receptors (5) having an extracellular single–chain antibody fragment as a tumor antigen–binding unit.

Recently, we have developed a fully synthetic cytokine/cytokine receptor system that mimicked natural cytokine signaling, exemplified by the proinflammatory cytokines interleukin (IL-)6, IL-12, IL-22, IL-23, tumor necrosis factor (TNF)α, and death ligand Fas (6, 7, 8, 9). This fully synthetic cytokine receptor system (SyCyR) was based on nanobodies specifically recognizing GFP and mCherry (10, 11) fused to the transmembrane and intracellular domains of the receptor of interest. A nanobody or VHH consists of the N-terminal variable domain of Camelidae heavy–chain antibody, which is sufficient for antigen binding (12). Nanobodies are already used in diagnostic and therapeutic applications and are immunologically safe (13). GFP-mCherry fusion proteins served as nanobody-cytokine receptor dimerizers (9). GFP and mCherry as synthetic cytokine ligands and specific nanobodies as receptor entities enabled background-free and cell-type–specific activation of synthetic cytokine receptors due to the lack of existing human equivalents for these antigen–antibody interactions. However, the nonhuman nature of GFP/mCherry represents a significant drawback because repetitive therapeutic application will lead to the development of neutralizing antibodies (14), which eventually will prevent receptor binding of synthetic ligands. Therefore, we considered an alternative synthetic cytokine/cytokine receptor pair, which should be based on a nanobody as extracellular receptor moiety. This nanobody should not bind to human proteins, and the synthetic ligand should not be immunogenic in humans. We considered that an antibody:anti-idiotypic nanobody pair in which the antibody is not directed against a human protein might be suited for this purpose. Moreover, the antibody should be licensed for human therapeutic applications to ease later approval.

Anti-idiotypes are antibodies or nanobodies that bind specifically to the variable regions or hypervariable loops of an antibody (15). Accordingly, we selected Palivizumab as bait for the development of anti-idiotypic nanobodies. Palivizumab is a monoclonal humanized antibody (IgG) directed against an epitope in the antigenic site of the fusion (F) protein of respiratory syncytial virus (RSV) (16). It was approved in 1998 to prevent infection and severe disease caused by RSV in infants at high risk. Palivizumab inhibits the entry of RSV into host cells (16). We immunized a llama with Palivizumab, followed by the selection of anti-idiotypic nanobodies using yeast display technology. Palivizumab and reformatted dimeric Palivizumab single–chain variable fragment (P^scFv^) served as synthetic cytokine ligands to activate the synthetic anti-idiotypic Palivizumab nanobody (AIP^VHH^) cytokine receptor fusion protein.

## Results

### Generation and characterization of anti-idiotypic nanobodies against Palivizumab (AIP^VHH^)

**Figure 1:**
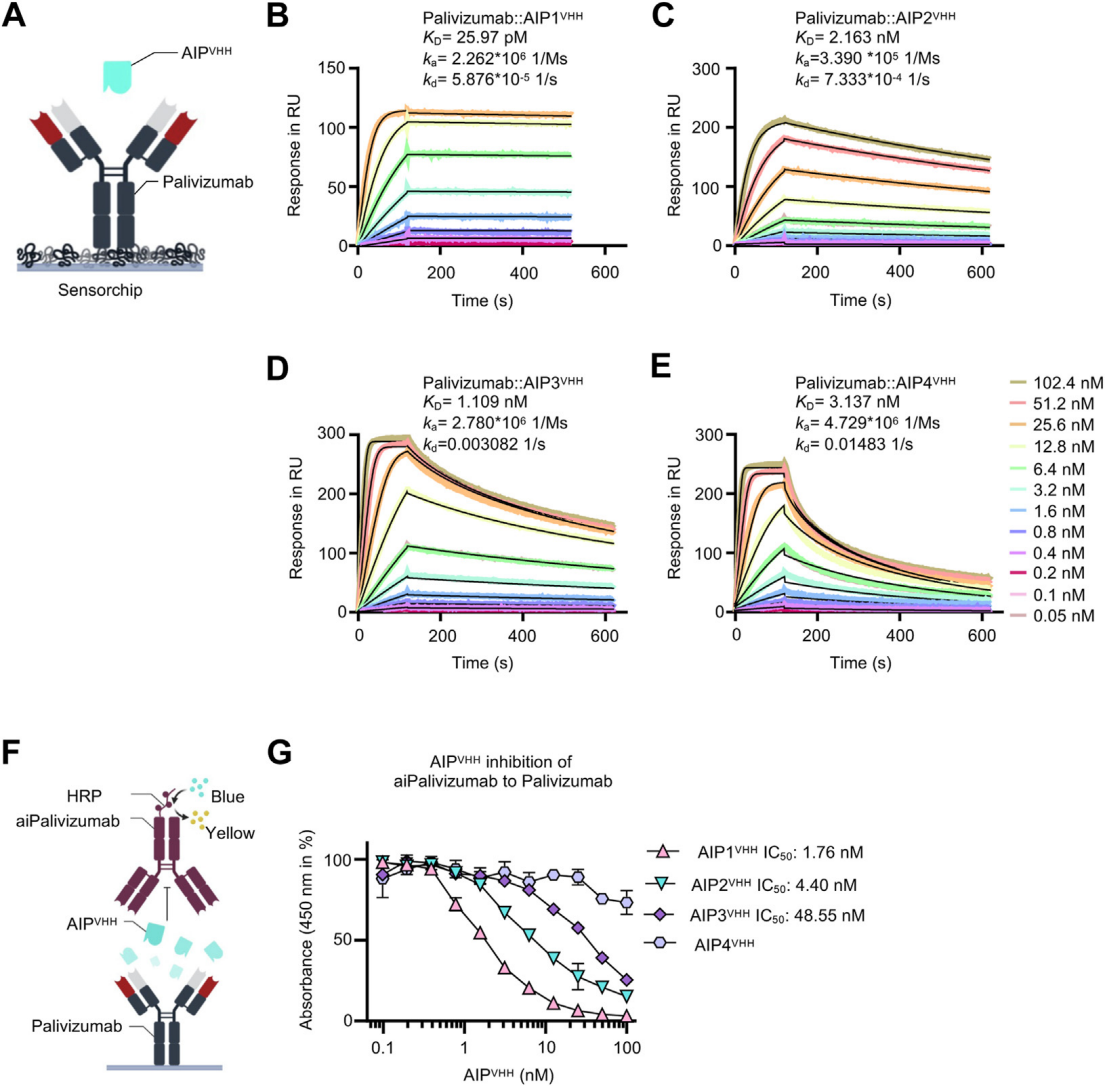
**Characterization of anti-idiotypic nanobodies against Palivizumab.***A*, schematic illustration of surface plasmon resonance analytes with captured Palivizumab coated on a Protein A chip and soluble AIP^VHH^. Surface plasmon data from binding of (*B*) AIP1^VHH^, (*C*) AIP2^VHH^, (*D*) AIP3^VHH^, and (*E*) AIP4^VHH^ (concentration range: 102.4, 51.2, 25.6, 12.8, 6.4, 3.2, 1.6, 0.8, 0.4, 0.2, 0.1, and 0.05 nM) to Palivizumab. Sensograms in response units (RU) over time are depicted as *colored lines*, global fit displayed as *black lines*. Analytes were injected for 120 s and dissociation rate was recorded for 500 s. *F*, schematic illustration of the competitive anti-idiotypic Palivizumab ELISA. Palivizumab was coated and binding of aiPalivizumab is quantified, coincubation with competitors AIP1-4^VHH^ should reduce signal strength. *G*, competitive anti-idiotypic Palivizumab ELISA. Incubation of 1 nM anti-idiotypic Palivizumab IgG antibody (AbD23967) as detection antibody with increasing amounts of soluble AIP1-4^VHH^ nanobodies (0.1–100 nM). Error bars, SD. One representative experiment with three biological replicates is shown (n = 3). AIP, anti-idiotypic nanobodies for Palivizumab.

A llama was immunized with Palivizumab, peripheral B cells were isolated, copy DNAs (cDNAs) coding for the VHH nanobody repertoire were amplified by PCR and introduced by gap repair cloning into linearized yeast display vector pCT using yeast strain EBY100 (17). Yeast cells were incubated with Palivizumab for flow cytometry sorting (Fig. S1*A*). Yeast cells were incubated with 1 mg/ml Gamunex 10% (human IgG mixture) to exclude unspecific IgG binders, followed by a fluorescent-labeled anti-human-fragment crystallizable (Fc)-phycoerythrin conjugate incubation. These prestained cells were incubated with Palivizumab (60 nM), followed by an anti-Fab(κ-chain)-allophycocyanin (APC) conjugate. Cells carrying only the APC fluorescence (Palivizumab-specific) were selected (Fig. S1*B*). After two rounds of sorting, clones were sequenced, and four different anti-idiotypic nanobodies for Palivizumab (AIP1-4^VHH^) were isolated (Fig. S2), expressed as Twin-Strep-tagged soluble proteins in Expi293F cells and purified by affinity chromatography (Fig. S3). First, the affinity of soluble AIP1-4^VHH^ to Palivizumab was determined by surface plasmon resonance (Fig. 1*A*). Soluble AIP1^VHH^ displayed a *K*_D_ of 25.97 pM (Fig. 1*B*), AIP2^VHH^ of 2.16 nM (Fig. 1*C*), AIP3^VHH^ of 1.11 nM (Fig. 1*D*), and AIP4^VHH^ of 3.14 nM (Fig. 1*E*) for Palivizumab, respectively. The monomeric AIP1^VHH^ revealed a very stable complex with Palivizumab characterized by a high *k*_a_ of 2.3 × 10^6^ 1/Ms and a low *k*_d_ of 5.9 × 10^−5^ 1/s. Next, we used the commercially available Palivizumab-specific anti-idiotypic IgG antibody AbD23967 (aiPalivizumab) to test for anti-idiotypic binding of AIP^VHH^ to Palivizumab, because AIP^VHH^ should compete for binding with aiPalivizumab to Palivizumab (Fig. 1*F*). Titration of AIP1^VHH^, AIP2^VHH^, and AIP3^VHH^ but not of AIP4^VHH^ resulted in a clear dose-dependent displacement of aiPalivizumab with an IC_50_ of 1.76 nM, 4.40 nM, and 48.55 nM, respectively (Figs. 1*G* and S4). Therefore, we conclude that AIP1^VHH^, AIP2^VHH^, and AIP3^VHH^ have the same or an overlapping anti-idiotypic binding site to Palivizumab as aiPalivizumab, while AIP4^VHH^ either has a too high *k*_d_ to compete with aiPalivizumab in this assay or it binds at a different epitope. Our data showed that AIP1-3^VHH^ are high-affinity anti-idiotypic binders to Palivizumab.

**Figure 2:**
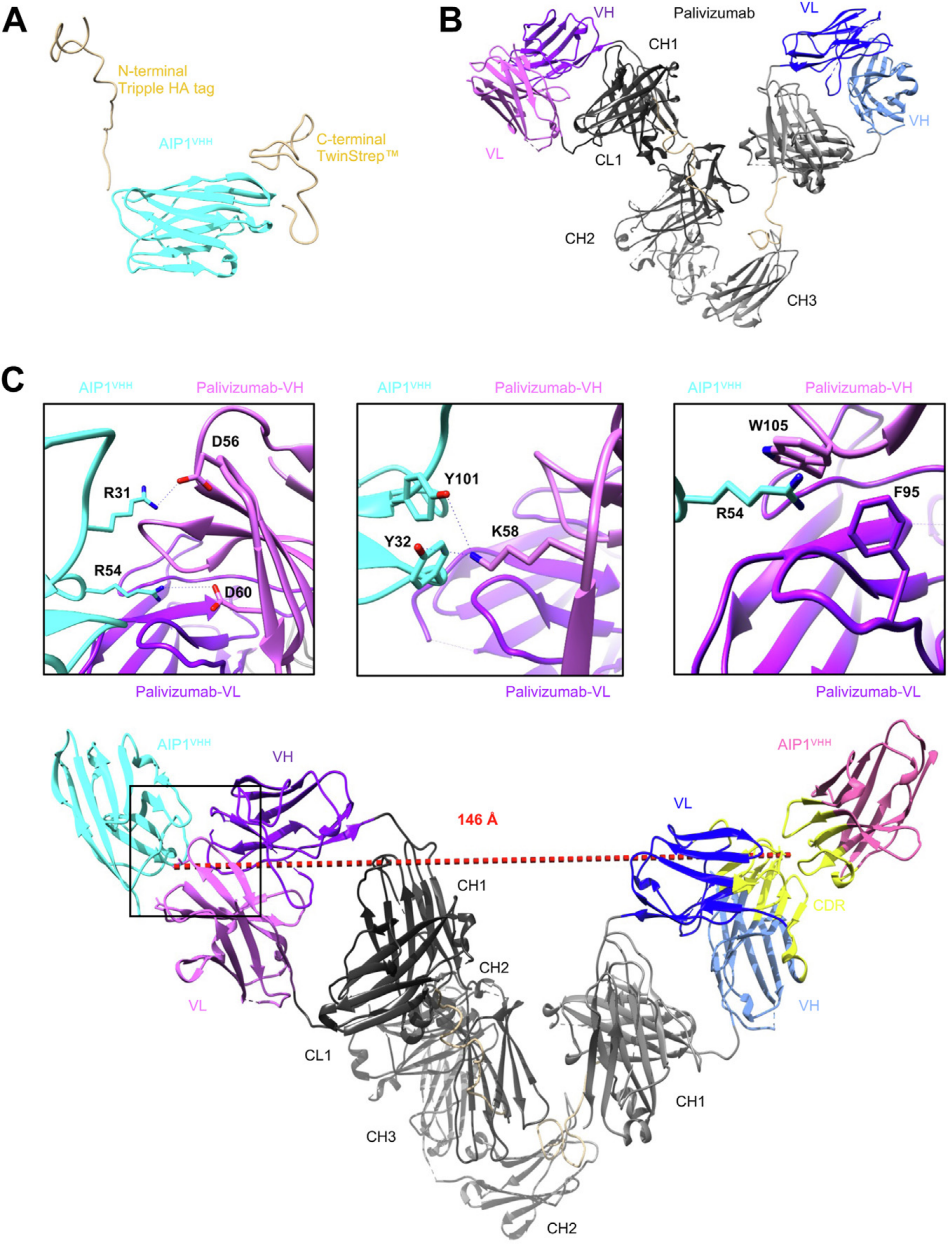
**Small-angle X-ray scattering structural characterization of the AIP1**^**VHH**^**and Palivizumab complex.***A*, rigid body model of AIP1^VHH^ based on AlphaFold from small-angle X-ray scattering with flexible N- and C-terminal parts. *B*, rigid body model of the IgG with flexible linkers (*beige*) between the Fc and Fab domains of Palivizumab. *C*, docking of AIP1^VHH^ to Palivizumab. Distance between the paratopes is 146 Å and is indicated by a *red dotted line*. On the *right side*, residues of AIP1^VHH^ and Palivizumab CDRs are colored *yellow*. Close-up views of the interaction area highlight hot spot amino acids, distances are indicated with *blue dotted lines*. Heavy chain, *purple* or *dark blue*; light chain, *pink* or *light blue*; constant region, *black* and *coal*; and AIP1^VHH^, *cyan* or *magenta*. AIP, anti-idiotypic nanobodies for Palivizumab; CDR, complementarity determining region; Fab, fragment antigen–binding; Fc, fragment crystallizable.

To further determine the anti-idiotypic character of AIP1^VHH^, we determined the small-angle X-ray scattering (SAXS) profile of AIP1^VHH^ (Table S1 and Fig. S6), Palivizumab (Table S1 and Fig. S7, *A*, *C*, and *E* black curves), and the complex of both (Table S1 and Fig. S7, *B*, *C*, and *E* green curves) (18, 19, 20, 21, 22). The AlphaFold2 models of the Fc and Fab fragments were used as template and subsequently realigned with CORAL until the protein tertiary structure describe the SAXS profile. We show that AIP1^VHH^ (Fig. 2*A*) and Palivizumab (Fig. 2*B*) are folded correctly and AIP1^VHH^ is a monomer and Palivizumab a dimer in solution (Table S1). The incubation of AIP1^VHH^ and Palivizumab results in a complex, where each monomer of Palivizumab binds one AIP1^VHH^. As expected AIP1^VHH^ binds the hypervariable antigen–binding loops of Palivizumab and sterically covers the loops on both heavy and light chain (Fig. 2*C*). We determined an important role between R31/R54 and Y101/Y32 of AIP1^VHH^ and D56/D60 and K58 of Palivizumab light chain, respectively. Furthermore, we assume hydrophobic interactions of W105 and F95 of Palivizumab with R54 of AIP1^VHH^ *via* cation–π interactions (Fig. 2*C*) (23).

### Cross-linked Palivizumab efficiently activated synthetic AIP^VHH^-gp130 receptor signaling

**Figure 3:**
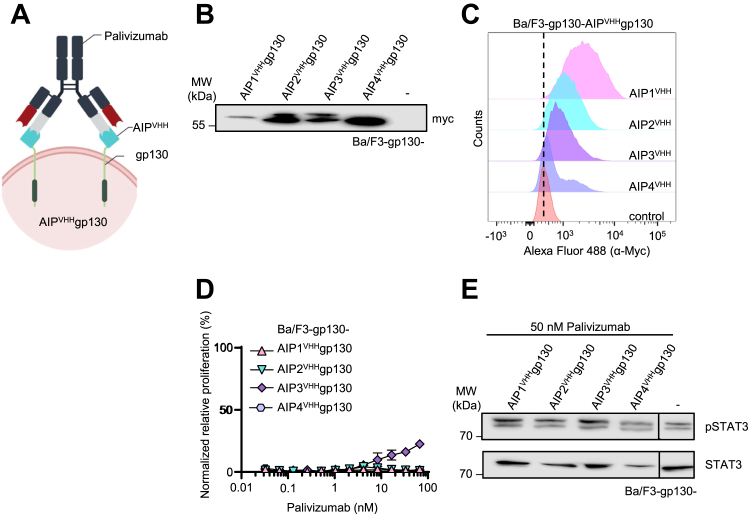
**Palivizumab is a poor activator of synthetic AIP3**^**VHH**^**gp130 receptor signaling.***A*, schematic illustration of Palivizumab binding to cellular AIP^VHH^gp130. *B*, Western blot detection of myc-tagged synthetic cytokine receptors in lysates of Ba/F3-gp130 cell lines expressing AIP1-4^VHH^gp130. *C*, flow cytometry analysis of myc-tagged synthetic receptors on the surface of Ba/F3-gp130 cells expressing AIP1-4^VHH^gp130. *D*, proliferation of Ba/F3-gp130 cells expressing AIP1-4^VHH^gp130 with increasing concentrations of Palivizumab (0.033–66 nM). Cell proliferation was normalized to HIL-6 (10 ng/ml)–induced proliferation of each cell line. Error bars, SD. One representative experiment with three biological replicates out of three independent experiments is shown. *E*, STAT3 phosphorylation in Ba/F3-gp130 cells expressing AIP1-4^VHH^gp130 cells treated with 50 nM Palivizumab, HIL-6 (10 ng/ml), or left untreated for 90 min. Equal amounts of proteins (50 μg/lane) were analyzed *via* specific antibodies detecting phospho-STAT3 and STAT3. Western blotting data show one representative experiment out of three. AIP, anti-idiotypic nanobodies for Palivizumab; HIL, hyper interleukin; STAT3, signal transducer and activator of transcription 3.

**Figure 4:**
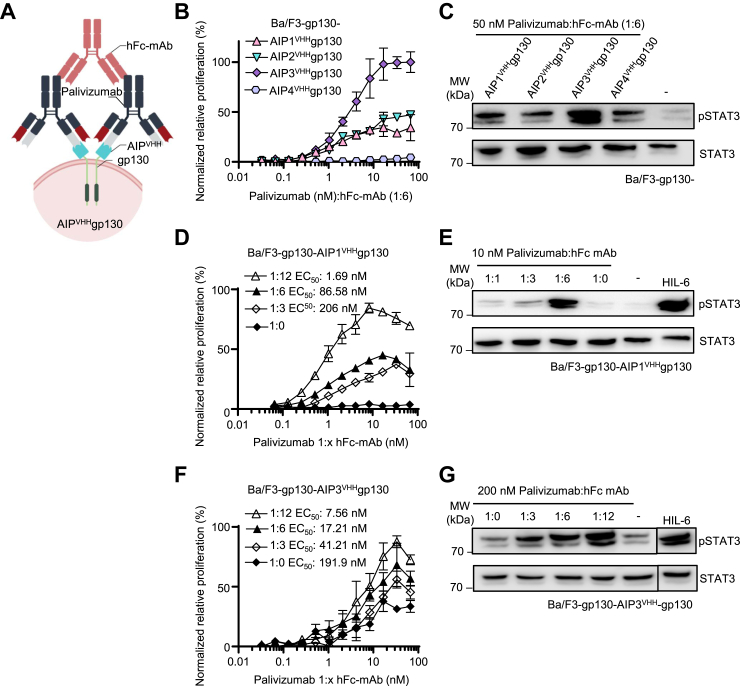
**Cross-linked Palivizumab efficiently activated synthetic AIP1-3**^**VHH**^**gp130 receptor signaling.***A*, schematic illustration of cross-linked Palivizumab binding to cellular AIP^VHH^gp130. Cross-linking is achieved by a Palivizumab Fc–binding mAb. *B*, proliferation of Ba/F3-gp130 cells expressing AIP1-4^VHH^gp130 with increasing concentrations of Palivizumab (0.033–66 nM) in the presence of a 6-fold molar excess of hFc-mAb. Cell proliferation was normalized to HIL-6 (10 ng/ml)–induced proliferation of each cell line. Error bars, SD. One representative experiment with three biological replicates out of three independent experiments is shown. *C*, STAT3 phosphorylation in Ba/F3-gp130 cells expressing AIP1-4^VHH^gp130 cells treated with 50 nM Palivizumab in the presence of a 6-fold molar excess of hFc-mAb, HIL-6 (10 ng/ml), or left untreated for 90 min. Equal amounts of proteins (50 μg/lane) were analyzed *via* specific antibodies detecting phospho-STAT3 and STAT3. Western blotting data show one representative experiment out of three. *D*, proliferation of Ba/F3-gp130 cells expressing AIP1^VHH^gp130 with increasing concentrations of Palivizumab (0.033–66 nM) in the presence of a 3-, 6-, and 12-fold molar excess of hFc-mAb. Cell proliferation was normalized to HIL-6 (10 ng/ml)–induced proliferation of each cell line. Error bars, SD. One representative experiment with three biological replicates out of three independent experiments is shown. *E*, STAT3 phosphorylation in Ba/F3-gp130 cells–expressing AIP1^VHH^gp130 cells treated with 10 nM Palivizumab in the presence of a 1-, 3-, 6-, and 6-fold molar excess of hFc-mAb, HIL-6 (10 ng/ml), or left untreated for 90 min. Equal amounts of proteins (50 μg/lane) were analyzed *via* specific antibodies detecting phospho-STAT3 and STAT3. Western blotting data show one representative experiment out of three. *F*, proliferation of Ba/F3-gp130 cells–expressing AIP3^VHH^gp130 with increasing concentrations of Palivizumab (0.033–66 nM) in the presence of a 3-, 6-, and 12-fold molar excess of hFc-mAb. Cell proliferation was normalized to HIL-6 (10 ng/ml)–induced proliferation of each cell line. Error bars, SD. One representative experiment with three biological replicates out of three independent experiments is shown. *G*, STAT3 phosphorylation in Ba/F3-gp130 cells expressing AIP3^VHH^gp130 cells treated with 200 nM Palivizumab in the presence of a 3-, 6-, 12-fold molar excess of hFc-mAb, HIL-6 (10 ng/ml), or left untreated for 90 min. Equal amounts of proteins (50 μg/lane) were analyzed *via* specific antibodies detecting phospho-STAT3 and STAT3. Western blotting data show one representative experiment out of three. AIP, anti-idiotypic nanobodies for Palivizumab; Fc, fragment crystallizable; hFC-mAb, human Fc–directed mAb; HIL, hyper interleukin; STAT3, signal transducer and activator of transcription 3.

AIP1-4^VHH^ were genetically fused to a cDNA coding for the transmembrane and intracellular domain of gp130 and named AIP1^VHH^gp130, AIP2^VHH^gp130, AIP3^VHH^gp130, and AIP4^VHH^gp130 (Figs. 3*A* and S5). The expression of AIP^VHH^gp130 proteins in Ba/F3-gp130 cells was shown by Western blotting against the N-terminal myc tag (Fig. 3*B*). Flow cytometry revealed that cell surface localization of AIP1^VHH^gp130 in Ba/F3-gp130 cells was stronger than AIP2^VHH^gp130 and AIP3^VHH^gp130, whereas AIP4^VHH^gp130 was the lowest, the expression levels might have an influence on the concentrations necessary for proliferation and might contribute to the distinct behavior of the receptors on the cells (Fig. 3*C*). Ba/F3-gp130 cells are murine pre-B cells, and due to stable expression of gp130, these cells become responsive to Hyper IL–6 (HIL-6, fusion protein of IL-6 and soluble IL-6 receptor α), which induces cell proliferation *via* Janus kinase (JAK)/signal transducer and activator of transcription (STAT) signaling (24). We expected that forced dimerization of the AIP^VHH^gp130 receptor by Palivizumab would result in synthetic cytokine receptor activation, induction of signal transduction, and cellular proliferation. Only minimal proliferation of Ba/F3-gp130 cells expressing AIP3^VHH^gp130 was observed, albeit the highest concentration of 66 nM Palivizumab induced only 20% of the maximal proliferation seen for 140 pM HIL-6. In contrast, Ba/F3-gp130 cells expressing AIP1,2,4^VHH^gp130 did not proliferate in response to Palivizumab (Fig. 3*D*). Palivizumab also failed to induce STAT3 phosphorylation in Ba/F3-gp130 cells expressing any of the AIP^VHH^gp130 receptors (Fig. 3*E*). We calculated the maximal distance between the two antigen-binding epitopes of Palivizumab and AIP1^VHH^ to be 150 Å using SAXS-based tertiary structure modeling (Fig. 2*C*). We hypothesized that this distance might be too spacious to activate two synthetic gp130 receptors. Therefore, higher ordered multimerization *via* a cross-linking human Fc–directed mAb (hFc-mAb) was tested to force synthetic gp130 receptor multimerization (Fig. 4*A*). Initially, we chose a 6-fold molar excess of the cross-linking hFc-mAb over Palivizumab to stimulate Ba/F3-gp130-AIP^VHH^gp130 cells. Cellular proliferation was observed for Ba/F3-gp130-AIP1^VHH^gp130 (EC_50_ = 215 nM), AIP2^VHH^gp130 (EC_50_ = 48.78 nM) and AIP3^VHH^gp130 (EC_50_ = 2.71 nM) cells but not for Ba/F3-gp130-AIP4^VHH^gp130 cells at the highest concentration of Palivizumab/hFc-mAb (Fig. 4*B*). As shown in Figure 4*C*, stimulation with 50 nM Palivizumab/hFc-mAb (1:6) induced STAT3 phosphorylation in all four Ba/F3-gp130-AIP^VHH^gp130 cell lines, whereas the intensity was strongest for Ba/F3-gp130-AIP1^VHH^gp130 and Ba/F3-gp130-AIP3^VHH^gp130. Neither the unrelated IL-23_p40 antibody Ustekinumab in combination with the cross-linking hFc-mAb (1:6) nor the cross-linking hFc-mAb alone did induce STAT3 phosphorylation and proliferation of any Ba/F3-gp130-AIP^VHH^gp130 cell line (Fig. S9, *A*–*C*). Next, the ratio of Palivizumab:hFc-mAb was varied from 1:0, 1:3, 1:6, to 1:12 (Fig. 4, *D*–*G*) and tested on Ba/F3-gp130-AIP1/3^VHH^gp130 cells. Proliferation was already induced by a 1:3 Palivizumab:hFc-mAb ratio. However, a 1:12 ratio was the most efficient (Fig. 4, *D* and *F*). Moreover, stimulation of the Ba/F3-gp130-AIP1/3^VHH^gp130 cells with Palivizumab:hFc-mAb from 1:3 to 1:12 M ratios resulted in sustained STAT3 phosphorylation (Fig. 4, *E* and *G*).

Taken together, our data showed that cross-linked Palivizumab efficiently activated the synthetic AIP^VHH^gp130 cytokine receptors AIP1,2,3^VHH^.

### Reformatting of Palivizumab into single-chain Fv fragments, maintained binding to AIP1^VHH^

**Figure 5:**
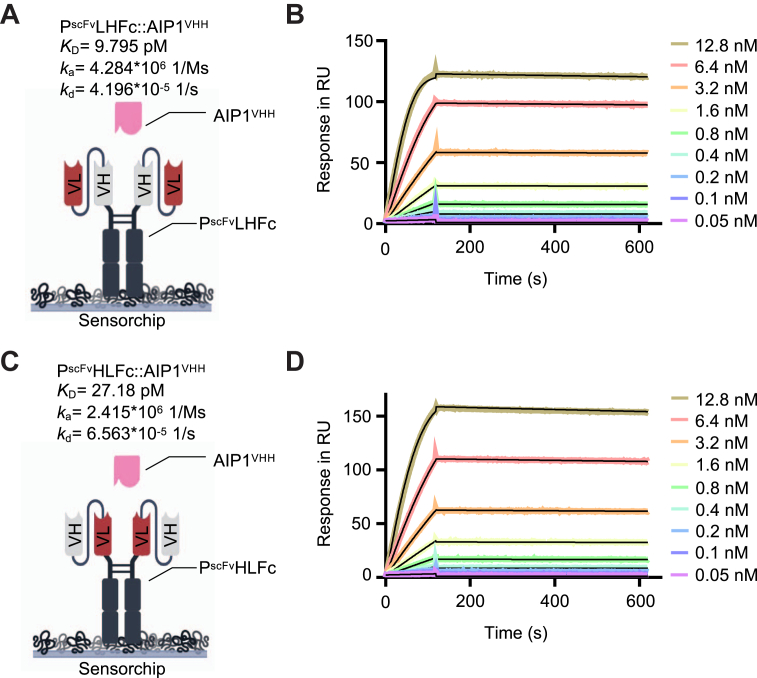
**Reformatting of Palivizumab into P**^**scFv**^**maintained binding to AIP1**^**VHH**^**.** Schematic illustration and surface plasmon resonance data with captured (*A* and *B*) P^scFv^LHFc and (*C* and *D*) P^scFv^HLFc coated on a Protein A chip and soluble AIP1^VHH^ (concentration range: 12.8, 6.4, 3.2, 1.6, 0.8, 0.4, 0.2, 0.1, and 0.05 nM). Sensograms in response units (RU) over time are depicted as *colored lines*, global fit displayed as *black lines*. Analytes were injected for 120 s and dissociation rate was recorded for 500 s. AIP, anti-idiotypic nanobodies for Palivizumab; P^scFv^, Palivizumab single–chain variable fragment.

Palivizumab alone is a poor activator of synthetic AIP^VHH^gp130 receptors, which might be due to the hinge region flexibility and the determined distance of about 150 Å between the two antigen-binding sites (Fig. 2*C*). Therefore, we reformatted Palivizumab into single-chain Fv fragments where the variable domains of the light and the heavy chain were fused by a flexible peptide linker, resulting in the cDNAs coding for P^scFv^LH and P^scFv^HL. The designation LH and HL indicated the order of the variable domains of the light chain (L) or the heavy chain (H). In LH, the variable domain of the light chain is N terminally located, whereas in HL, the variable domain of the heavy chain is N terminally located (Fig. S8*A*). P^scFv^LH and P^scFv^HL were fused to an Fc part of an IgG1 antibody (25). The distance of P^scFv^LH or P^scFv^HL to the first N-terminal cysteine of the Fc hinge region was 23 and 15 amino acids, respectively. Further, the linker ensures a maximal distance of the variable domains of about 90 and 114 Å as defined by molecular modeling (Fig. S8, *A* and *B*). P^scFv^LHFc and P^scFv^HLFc were expressed in stably transfected CHO cells and transiently transfected Expi293F cells, respectively, and purified as dimers from the cell supernatants *via* Protein A affinity chromatography (Fig. S10, *A*–*F*). Next, we determined the interaction affinities of captured P^scFv^LHFc and P^scFv^HLFc to soluble AIP1^VHH^ by surface plasmon resonance. AIP1^VHH^ binds P^scFv^LHFc, P^scFv^HLFc, and Palivizumab with comparable affinities of 9.80 pM, 27.18 pM, and 25.97 pM, respectively (Figs. 1*B* and 5), demonstrating successful reformatting of Palivizumab into scFv fragments.

### P^scFv^Fc are effective activators of synthetic AIP^VHH^gp130 receptors

**Figure 6:**
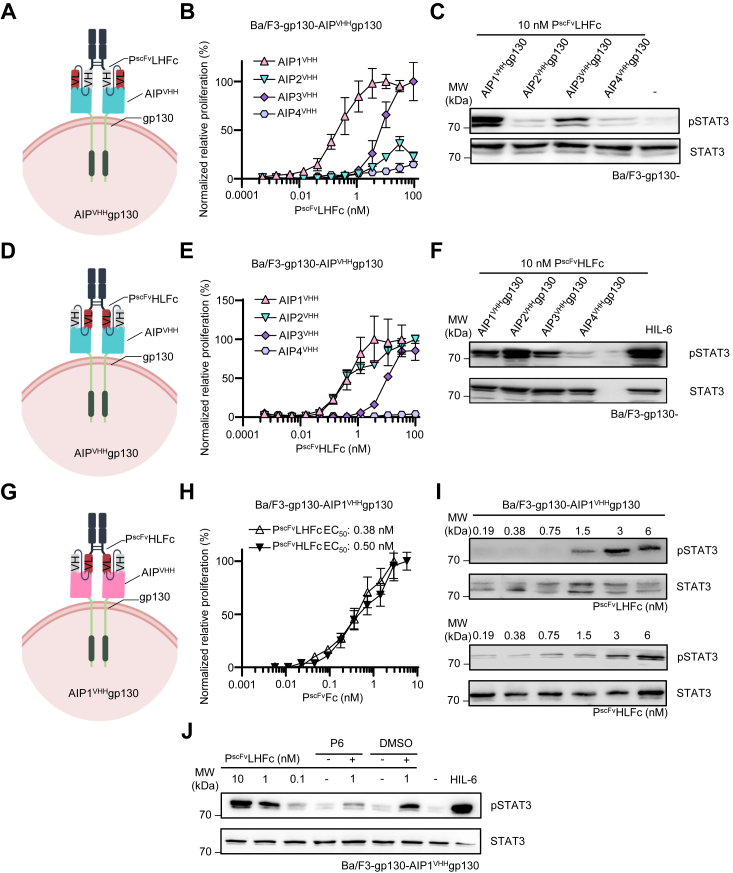
**P**^**scFv**^**Fc are effective activators of synthetic AIP**^**VHH**^**gp130 receptors.***A*, schematic illustration of P^scFv^LHFc binding to cellular AIP^VHH^gp130. *B*, proliferation of Ba/F3-gp130 cells expressing AIP1-4^VHH^gp130 with increasing concentrations of P^scFv^LHFc (0.00053–93.5 nM). Cell proliferation was normalized to HIL-6 (10 ng/ml) induced proliferation of each cell line. Error bars, SD. One representative experiment with three biological replicates out of three independent experiments is shown. *C*, STAT3 phosphorylation in Ba/F3-gp130 cells expressing AIP1-4^VHH^gp130 cells treated with 10 nM P^scFv^LHFc or left untreated for 90 min. *D*, schematic illustration of P^scFv^HLFc binding to cellular AIP^VHH^gp130. *E*, proliferation of Ba/F3-gp130 cells expressing AIP1-4^VHH^gp130 with increasing concentrations of P^scFv^HLFc (0.00056–100 nM). Cell proliferation was normalized to HIL-6 (10 ng/ml)–induced proliferation of each cell line. *F*, STAT3 phosphorylation in Ba/F3-gp130 cells expressing AIP1-4^VHH^gp130 cells treated with 10 nM P^scFv^HLFc or HIL-6 (10 ng/ml) for 90 min. *G*, schematic illustration of P^scFv^LHFc, and P^scFv^HLFc binding to cellular AIP1^VHH^gp130. *H*, proliferation of Ba/F3-gp130 cells expressing AIP1^VHH^gp130 with increasing concentrations of P^scFv^LHFc and P^scFv^HLFc (0.00563–5.77 nM). Cell proliferation was normalized to HIL-6 (10 ng/ml) induced proliferation of each cell line. Error bars, SD. One representative experiment with three biological replicates out of three independent experiments is shown. *I*, phosphorylation of STAT3 in Ba/F3-gp130-AIP1^VHH^gp130 cells treated with increasing concentrations of P^scFv^LHFc or P^scFv^HLFc from 0.19 to 6 nM or left untreated for 90 min. Equal amounts of proteins (50 μg/lane) were analyzed *via* specific antibodies detecting phospho-STAT3 and STAT3. Western blotting data show one representative experiment out of three. *J*, phosphorylation of STAT3 in Ba/F3-gp130-AIP1^VHH^gp130 cells treated with 0.1, 1, and 10 nM P^scFv^LHFc in the presence and absence of 10-μm P6 inhibitor, HIL-6 (10 ng/ml), or left untreated for 90 min. Dimethyl sulfoxide (1% v/v)-treated cells served as control. AIP, anti-idiotypic nanobodies for Palivizumab; Fc, fragment crystallizable; HIL, hyper interleukin; P^scFv^, Palivizumab single–chain variable fragment; STAT3, signal transducer and activator of transcription 3.

Next, we tested P^scFv^LHFc on Ba/F3-gp130-AIP^VHH^gp130 cells to induce cell proliferation and STAT3 phosphorylation (Fig. 6*A*). As only seen for cross-linked Palivizumab, P^scFv^LHFc alone induced cellular proliferation and STAT3 phosphorylation *via* AIP^VHH^gp130. Dose-dependent stimulation of Ba/F3-gp130 cells expressing AIP^VHH^gp130 with P^scFv^LHFc revealed an EC_50_ of 0.21 nM for AIP1^VHH^gp130 and 6.61 nM for AIP3^VHH^gp130 (Fig. 6*B*), which is in good agreement with natural cytokine concentrations needed for receptor activation (6). However, the expression of AIP2^VHH^gp130 was the least effective, and AIP4^VHH^gp130 failed to induce cellular proliferation (Fig. 6*B*). These findings were mirrored by STAT3 phosphorylation, where 10 nM P^scFv^LHFc was most effective on AIP1^VHH^gp130, followed by AIP3^VHH^gp130 (Fig. 6*C*). AIP2^VHH^gp130 and AIP4^VHH^gp130 were not activated by P^scFv^LHFc (Fig. 6*C*). Next, we tested whether the alternative heavy/light chain ordered single-chain P^scFv^HLFc is also biologically active (Fig. 6*D*). The activity of P^scFv^HLFc was determined for Ba/F3-gp130-AIP1^VHH^gp130 and Ba/F3-gp130-AIP3^VHH^gp130 cells with EC_50_ of 0.4 nM and 9.1 nM, respectively. In contrast, cellular proliferation of Ba/F3-gp130-AIP2^VHH^gp130 was induced with P^scFv^HLFc with an EC_50_ of 0.7 nM (Fig. 6*E*). Stimulation with 10 nM P^scFv^HLFc induced STAT3 phosphorylation in Ba/F-3-gp130-AIP1-3^VHH^gp130 cells, whereas the intensity was strongest for Ba/F3-gp130-AIP2^VHH^gp130, followed by Ba/F3-gp130-AIP1^VHH^gp130, and AIP3^VHH^gp130 (Fig. 6*F*). We found that P^scFv^HLFc induced proliferation of Ba/F3-gp130-AIP1^VHH^gp130 cells with an EC_50_ of 0.50 nM (Fig. 6*H*), which was in the same range as seen for P^scFv^LHFc (EC_50_ of 0.38 nM). Accordingly, low amounts starting at 0.19 nM P^scFv^HLFc and 1.5 nM P^scFv^LHFc already induced STAT3 phosphorylation of Ba/F3-gp130-AIP1^VHH^gp130 cells (Fig. 6*I*). STAT3 phosphorylation in Ba/F3-gp130-AIP1^VHH^gp130 cells was suppressed by the pan JAK inhibitor P6 (26), demonstrating that STAT3 activation was mediated *via* the JAK/STAT pathway, following activation of AIP1^VHH^gp130 (Fig. 6*J*).

**Figure 7:**
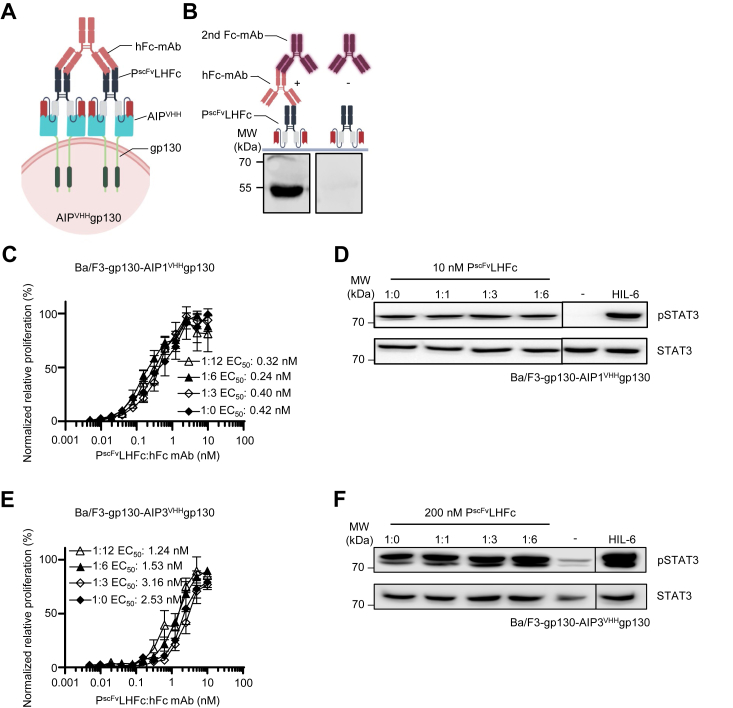
**Cross-linking of P**^**scFv**^**Fc did not enhance synthetic AIP**^**VHH**^**gp130 receptor activation.***A*, schematic illustration of cross-linked P^scFv^LHFc binding to cellular AIP^VHH^gp130. Cross-linking is achieved by a P^scFv^LHFc Fc–binding mAb. *B*, Western blotting of P^scFv^LHFc with and without primary Fc-binding mAb from goat (hFc-mAb, used for cross-linking), followed by secondary anti-goat specific antibody (second Fc-mAb). *C*, proliferation of Ba/F3-gp130 cells expressing AIP1^VHH^gp130 with increasing concentrations of P^scFv^LHFc (0.0049–10 nM) in the presence of a 3-, 6-, and 12-fold molar excess of hFc-mAb. Cell proliferation was normalized to HIL-6 (10 ng/ml)–induced proliferation of each cell line. Error bars, SD. One representative experiment with three biological replicates out of three independent experiments is shown. *D*, STAT3 phosphorylation in Ba/F3-gp130 cells expressing AIP1^VHH^gp130 cells treated with 10 nM P^scFv^LHFc in the presence of a 1-, 3-, and 6-fold molar excess of hFc-mAb, HIL-6 (10 ng/ml), or left untreated for 90 min. Equal amounts of proteins (50 μg/lane) were analyzed *via* specific antibodies detecting phospho-STAT3 and STAT3. Western blotting data show one representative experiment out of three. *E*, proliferation of Ba/F3-gp130 cells expressing AIP3^VHH^gp130 with increasing concentrations of P^scFv^LHFc (0.0049–10 nM) in the presence of a 3-, 6-, and 12-fold molar excess of hFc-mAb. Cell proliferation was normalized to HIL-6 (10 ng/ml)–induced proliferation of each cell line. Error bars, SD. One representative experiment with three biological replicates out of three independent experiments is shown. *F*, STAT3 phosphorylation in Ba/F3-gp130 cells expressing AIP3^VHH^gp130 cells treated with 200 nM P^scFv^LHFc in the presence of a 1-, 3-, 6-fold molar excess of hFc-mAb, HIL-6 (10 ng/ml), or left untreated for 90 min. Equal amounts of proteins (50 μg/lane) were analyzed *via* specific antibodies detecting phospho-STAT3 and STAT3. Western blotting data show one representative experiment out of three. AIP, anti-idiotypic nanobodies for Palivizumab; Fc, fragment crystallizable; hFC-mAb, human Fc–directed mAb; HIL, hyper interleukin; P^scFv^, Palivizumab single–chain variable fragment; STAT3, signal transducer and activator of transcription 3.

Palivizumab-induced signaling was potentiated by cross-linking. Therefore, we tested the ability to induce receptor activation through cross-linking of P^scFv^LHFc with hFc-mAb (Fig. 7*A*). First, we showed that the hFc-mAb binds P^scFv^LHFc. The detection of P^scFv^LHFc was only achieved in the presence but not in the absence of hFc-mAb using Western blotting (Fig. 7*B*). Increasing the molar ratio of hFc-mAb:P^scFv^LHFc from 1:0, 1:3, 1:6, to 1:12 did, however, not enhance the proliferation of Ba/F3-gp130-AIP1/3^VHH^gp130 cells (Fig. 7, *C* and *E*). Cross-linking did also not increase STAT3 phosphorylation of Ba/F3-gp130-AIP1/3^VHH^gp130 cells (Fig. 7, *D* and *F*). As a control, neither Palivizumab nor P^scFv^LHFc alone induced cellular proliferation or STAT3 phosphorylation of Ba/F3-gp130 cells (Fig. S9, *D* and *E*).

In summary, the reformatted dimeric P^scFv^ variants are efficient anti-idiotypic synthetic cytokine ligands that induce sustained signal transduction and cell proliferation. Cross-linking did, however, not increase the activity of the synthetic gp130 cytokine receptors.

### Competition of Palivizumab and P^scFv^ inhibits synthetic AIP1^VHH^gp130 signaling

**Figure 8:**
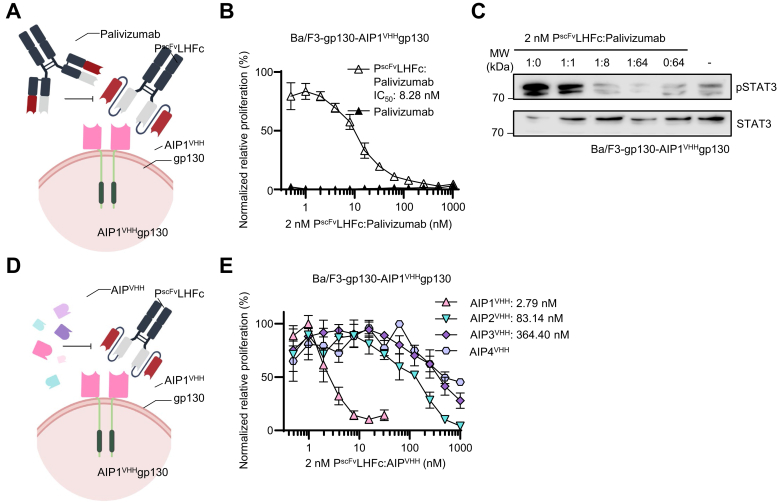
**Competition of Palivizumab and P**^**scFv**^**inhibits synthetic AIP1**^**VHH**^**gp130 signaling.***A*, schematic illustration of competition between Palivizumab and P^scFv^LHFc on Ba/F3-gp130-AIP1^VHH^gp130 cells. *B*, proliferation of Ba/F3-gp130 cells expressing AIP1^VHH^gp130 with 1.8 nM P^scFv^LHFc in the presence increasing concentrations of Palivizumab (0.49–1000 nM). Cell proliferation was normalized to HIL-6 (10 ng/ml). As negative control, Ba/F3-gp130-AIP1^VHH^gp130 cells were treated with Palivizumab alone. Error bars, SD. One representative experiment with three biological replicates out of three independent experiments is shown. *C*, STAT3 phosphorylation in Ba/F3-gp130 cells expressing AIP1^VHH^gp130 cells treated with 2 nM P^scFv^LHFc in the presence of a 0-, 1-, 8-, and 64-fold molar excess of Palivizumab or left untreated for 90 min. Equal amounts of proteins (50 μg/lane) were analyzed *via* specific antibodies detecting phospho-STAT3 and STAT3. Western blotting data show one representative experiment out of three. *D*, schematic illustration of competition between P^scFv^LHFc and soluble AIP1-4^VHH^ on Ba/F3-gp130-AIP1^VHH^gp130 cells. *E*, proliferation of Ba/F3-gp130 cells expressing AIP1^VHH^gp130 with 2 nM P^scFv^LHFc in the presence increasing concentrations of soluble AIP1-4^VHH^ (0.49–1000 nM). Cell proliferation was normalized to HIL-6 (10 ng/ml). Error bars, SD. One representative experiment with three biological replicates out of three independent experiments is shown. AIP, anti-idiotypic nanobodies for Palivizumab; Fc, fragment crystallizable; HIL, hyper interleukin; P^scFv^, Palivizumab single–chain variable fragment; STAT3, signal transducer and activator of transcription 3.

Both dimeric Palivizumab scFv variants P^scFv^LHFc and P^scFv^HLFc efficiently activate synthetic AIP1^VHH^gp130 cytokine receptor signaling. As depicted in Figure 8*A*, competition experiments with a constant concentration of P^scFv^LHFc and increasing concentrations of Palivizumab showed that Palivizumab is an inhibitor of P^scFv^LHFc-induced receptor activation. 2 nM P^scFv^LHFc was used to induce sustained proliferation of Ba/F3-gp130-AIP1^VHH^gp130 cells (Fig. 8*B*). Adding increasing amounts of Palivizumab resulted in dose-dependent suppression of cellular proliferation. The IC_50_ for inhibition of P^scFv^LHFc by Palivizumab was 8.28 nM, demonstrating 4-fold excess of Palivizumab was sufficient to inhibit P^scFv^LHFc-induced cell proliferation (Fig. 8*B*). For STAT3 phosphorylation, at least an 8-fold molar excess of Palivizumab over P^scFv^LHFc (2 nM) resulted in complete suppression of signal transduction in Ba/F3-gp130-AIP1^VHH^gp130 cells (Fig. 8*C*). Next, monomeric AIP^VHH^ was tested for inhibition of P^scFv^LHFc-induced proliferation of Ba/F3-gp130-AIP1^VHH^gp130 cells (Fig. 8*D*). Soluble AIP1^VHH^ inhibited P^scFv^LHFc (2 nM) induced cellular proliferation with an IC_50_ of 2.79 nM, whereas higher inhibitory concentrations of AIP2^VHH^ and AIP3^VHH^ were needed (IC_50_ = 83.14 and 364.40 nM, respectively). AIP4^VHH^ might not function as an antagonist because of its high *k*_d_ compared to AIP2^VHH^ and AIP3^VHH^ (Fig. 8*E*). The high affinity of AIP1^VHH^ might explain these inhibitory differences compared to AIP2^VHH^, AIP3^VHH^, and AIP4^VHH^ to Palivizumab.

### Synthetic AIP^VHH^Fas receptors efficiently induce cellular apoptosis

**Figure 9:**
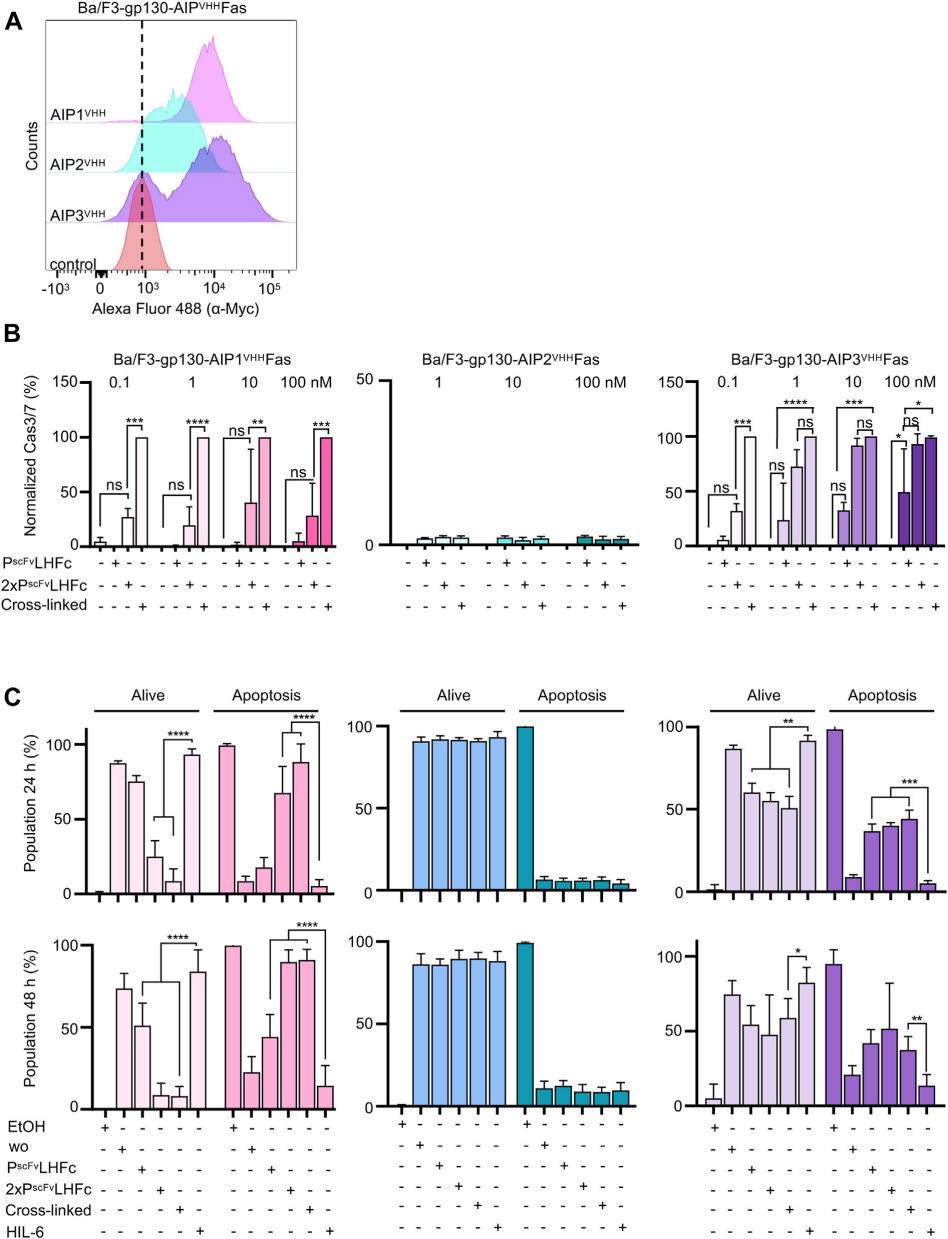
**AIP**^**VHH**^**fusion to Fas efficiently induce cellular apoptosis.***A*, flow cytometry analysis of myc-tagged synthetic receptors on the surface of Ba/F3-gp130 cells expressing AIP1-3^VHH^Fas. *B*, relative caspase-3/7 activity after incubation of Ba/F3-gp130 cells expressing AIP1-3^VHH^Fas with increasing concentrations (0.1, 1, 10, and 100 nM) of dimeric P^scFv^LHFc, tetrameric 2 × P^scFv^LHFc, or cross-linked 2 × P^scFv^LHFc for 6 h, control cells were left untreated. *C*, Ba/F3-gp130 cells expressing AIP1-3^VHH^Fas were incubated for 24 h or 48 h with 100 nM dimeric P^scFv^LHFc, tetrameric 2 × P^scFv^LHFc, or cross-linked 2 × P^scFv^LHFc (1:6). Controls cells were either untreated or grown with HIL-6 (10 ng/ml). Cells were incubated with HIL-6 (10 ng/ml) and washed with 70% EtOH before the measurement for the EtOH condition. Cells were stained with AnnexinV and 7-aminoactinomycin D and analysis was carried out using flow cytometry. AIP, anti-idiotypic nanobodies for Palivizumab; Fc, fragment crystallizable; hFC-mAb, human Fc–directed mAb; HIL, hyper interleukin; P^scFv^, Palivizumab single–chain variable fragment.

The death receptor Fas induces apoptosis through initiator Caspase 8 and effector Caspase 3/6/7 *via* the trimeric FasL (27, 28). Recent data, however, suggests also higher than trimeric receptor-oligomerization upon binding induce apoptosis (29). Using GFP/mCherry as synthetic ligands, we previously showed that apoptosis by the Fas-SyCyR was also induced by dimers, which was, however, less efficient than the trimeric or oligomeric Fas–SyCyR complexes (7). Here, the anti-idiotypic synthetic cytokine systems were adopted to Fas-induced apoptosis. AIP1-3^VHH^ were genetically fused to a cDNA coding for the transmembrane and intracellular domain of human Fas, with an N-terminal signal peptide, followed by a myc tag for detection (Fig. S5). Flow cytometry against the N-terminal myc tag showed cell surface expression of synthetic AIP1,2,3^VHH^Fas receptors on Ba/F3-gp130 cells (Fig. 9*A*). Ba/F3-gp130-AIP1-3^VHH^Fas cells were analyzed for activation of caspase 3/7 and induction of apoptosis after AIP^VHH^Fas stimulation in comparison to HIL6-induced gp130 activation. The synthetic dimeric P^scFv^LHFc and tetrameric 2× P^scFv^LHFc ligands were designed to induce dimeric and tetrameric AIP1-3^VHH^Fas receptor assemblies, respectively. Tetramerization of 2× P^scFv^LHFc was achieved by linker peptide connected tandem arrangement of two P^scFv^ fused to the IgG1-Fc fragment. Higher-ordered Fas oligomerization was induced by cross-linking of 2× P^scFv^LHFc with hFc-mAb (1:6 M ratio). Tetrameric and oligomeric ligands induced caspase 3/7 activation after 4 h in Ba/F3-gp130-AIP1^VHH^Fas and Ba/F3-gp130-AIP3^VHH^Fas cells (Fig. 9*B*). Apoptosis was not induced *via* AIP2^VHH^Fas incubated with any of the synthetic ligands. Compared to tetramerization, oligomerization of AIP1^VHH^Fas was about 2.5- to 5-fold more effective in inducing caspase 3/7. The amount of activated caspase 3/7 was maximal and independent of ligand concentration, when oligomerization clustered AIP1^VHH^Fas. For AIP3^VHH^Fas, 2× P^scFv^LHFc-induced caspase 3/7 activation upon tetramerization was as effective as oligomerization at least for 1, 10, and 100 nM. Additionally, dimeric P^scFv^LHFc activated AIP3^VHH^Fas up to 50% compared to oligomerization at 100 nM (Fig. 9*B*).

Finally, apoptosis of Ba/F3-gp130-AIP^VHH^Fas cells was quantified using flow cytometry after 24 and 48 h of stimulation with synthetic cytokine ligands. Ethanol treatment served as positive control, whereas stimulation with HIL-6 was the proliferation control (Fig. S11). Again, stimulation of AIP2^VHH^Fas in Ba/F3-gp130 cells failed to induce apoptosis, whereas Ba/F3-gp130 cells expressing AIP1^VHH^Fas and AIP3^VHH^Fas were apoptotic, following synthetic cytokine stimulation. Apoptotic Ba/F3-gp130 cells expressing AIP1^VHH^Fas were, however, also found after dimeric receptor activation with P^scFv^LHFc albeit not before 48 h, whereas tetrameric and oligomeric receptor activation efficiently induced apoptosis already after 24 h. Apoptotic Ba/F3-gp130 cells expressing AIP3^VHH^Fas were provoked after 24 h stimulation with dimeric, tetrameric, and oligomeric P^scFv^LHFc (100 nM) (Fig. 9*C*). Taken together, we have shown that activation of synthetic Fas efficiently induced apoptosis.

## Discussion

Our data showed that the generated antibody:anti-idiotypic nanobody-cytokine receptor pair is well-suited for synthetic cytokine receptor assembly and signaling. An anti-idiotypic antibody is directed against the hypervariable loops of another antibody, which constitute the antigen-binding site of the antibodies (15). In general, the fully synthetic cytokine/cytokine receptor system presented in this study results in cellular activation *via* gp130 or cellular apoptosis *via* Fas, which allows the assembly of up to four synthetic cytokine receptors in one complex. This system is on/off-switchable because signal activation can be rapidly inhibited by applying soluble nanobodies or Palivizumab, which opens up therapeutic regimes involving nonphysiological targets in immunotherapy. Reliably inducible death switches such as iCASP9 and our SyCyRs are anticipated in immunotherapy and may increase the safety index of CAR T-cell therapy (30, 31). We believe selecting an approved therapeutic antibody will enable the establishment of a from the shelf, nonimmunogenic, fully synthetic receptor system for the modulation, and selective activation of cytokine signaling in a complex environment without the danger of unwanted activation of neighboring nontarget cells. Recently, we developed a SyCyR based on nanobody/cytokine receptor fusion proteins activated by dimeric GFP and mCherry ligands (6). Moreover, we showed that the distance of the GFP/mCherry subunits in the synthetic dimeric ligand formed through Fc-mediated dimerization is crucial for effective receptor activation (9). This system was used to phenocopy cytokine signaling for IL-6 and IL-12 signaling, for IL-10 and interferon signaling, and also for the trimeric receptor class of tumor necrosis factor receptor/Fas (6, 7, 8, 32). The homomeric and heteromeric ligands generally showed high affinity and specificity. However, GFP and mCherry are foreign antigens to the body which eventually will produce antibodies, resulting in the neutralization of these synthetic ligands (14). Therefore, we envisioned that approved commercial antibodies might serve as surrogate synthetic ligands for anti-idiotypic nanobody-cytokine receptors. Our premise was that such antibodies should not have the human body´s targets or antigens. Therefore, clinically Food and Drug Administration (FDA)/European Medicines Agency (EMA)-approved antibodies with anti-inflammatory or anticancer properties, such as Tocilizumab (anti-IL-6R) (33), Infliximab (anti-TNFα) (34), or Ipilimumab (anti-CTLA-4) (35) were unsuitable because these target human proteins. Therefore, we identified several FDA/EMA-approved antibodies that target foreign viral and bacterial antigens, including anti-RSVs humanized IgG1 Palivizumab (16) and the follow-up human IgG1 Nirsevimab (RSV) (2), anti-SARS-CoV2 human IgG1s Sotrovimab (36), Regdanvimab (37), Imdevimab (38) or Casirivimab (38), anti-Ebola human IgG1 Ansuvimab (39) and the triple antibody cocktail REGN-EB3 (atoltivimab, maftivimab, odesivimab) (39), and anti-bacterial/anthrax human IgG1 Raxibacumab (40).

We chose Palivizumab to generate the first proof-of-principle anti-idiotypic nanobodies because Palivizumab has the longest-lasting positive safety history since its approval in 1998 (41). Palivizumab binds an epitope in the A antigenic site of the fusion (F) protein of RSV and inhibits the entry of RSV into host cells (16). Unfortunately, nanobody-cytokine receptor signaling was only efficiently activated after cross-linking Palivizumab by Fc antibodies. However, cross-linking of IgG antibodies remains challenging *in vivo*. Therefore, we reformatted Palivizumab in dimeric and tetrameric scFv-Fc fusion proteins, which were highly biologically active. We hypothesize that the distance of the variable regions/complementarity determining regions was too large for an efficient pairing of nanobody-cytokine receptors, which would likely be a common issue for other antibody classes. The overall structure of antibodies can be subdivided into the fragment antigen–binding (Fab) region and the Fc region. The Fc part is connected through interchain disulfide bridges, whereas the Fab region contains the variable domains, followed by one constant domain of the heavy or light chain in the IgG, IgD, and IgA antibody classes. In contrast, IgMs and IgEs contain two constant domains in Fab. In the scFv-Fc fusion proteins, which were the biologically active synthetic cytokine surrogates, the constant domains of the Fab region were deleted, thereby resulting in synthetic antibodies with closer variable domains (Fig. S8*B*). From the list of FDA/EMA-approved antibodies, all were IgG1 subtypes except Ibalizumab, Natalizumab, Lebrikizumab, Tislelizumab, Mirikizumab, and Gemtuzumab, as humanized IgG4 subtypes (42). It remains to be seen if reformatting Palivizumab into other antibody formats, such as IgA1/2, IgE, or IgM, will induce synthetic anti-idiotypic cytokine signaling without cross-linking. Since they also contain at least one constant domain within the Fab region, we assume that these antibody classes will likely not activate our synthetic cytokine receptors. IgMs might mimic the cross-linking effect due to the pentameric arrangement with ten antigen-binding sites. Therefore, reformatting Palivizumab scFv into an IgM antibody format lacking the CH1 and CL domains might be of interest in future studies. Reliable methods for their production have been established and the first IgM-derived antibodies have entered clinical trials (43).

The generation of further antibody/anti-idiotypic nanobody combinations of the listed anti-viral/bacterial antibodies might allow the assembly of heterodimeric receptor complexes. For now, heterodimeric receptor recruitment might also be possible with GFP or mCherry fused to Palivizumab scFv. We have not tested this possibility because this study generally aimed to replace GFP/mCherry as synthetic ligands. The tetrameric P^scFv^-P^scFv^-Fc fusion proteins enable the combination of two or more synthetic receptor pairs. It was shown in this study that Fas signaling activated apoptosis based on higher-ordered ligands. Notably, lower affinity between AIP^VHH^ and P^scFv^ leads to higher potency, in accordance with previous studies on TNF receptor superfamily signaling (44, 45). Alternatively, heterodimeric scFv can be generated with preferentially heterodimer-forming Fc through SEEDbodies or using the knob-into-hole technology for generating heavy-chain heterodimers (46, 47). Therefore, the generation of heterodimeric scFvs should be generally feasible. In case that other than IgG full-length antibody subtypes might serve as efficient synthetic ligands, these antibody subclasses might be used to generate bispecific antibodies as heterodimeric/multimeric synthetic ligands. The bispecific antibodies made significant progress in the last decade and many are in clinical development (48).

Both components used in this study can be considered nonimmunogenic since scFv is part of clinical applications' CAR T-cell therapy. Nanobodies have a high sequence similarity of 75 to 90% to the human VH3 gene family, explaining their general low immunogenicity in clinical applications (49), however, one or all of the AIP^VHH^s described in this study might be immunogenic nonetheless. Although the SyCyR system has been shown to function in mice (6), the presented antibody:anti-idiotypic nanobody-cytokine receptor pair has not been tested *in vivo* and might have to be optimized before use in clinical applications. While various nanobodies and scFvs are used in the clinic, no pharmacokinetic or pharmacovigilance analysis of the components used in this study has been done yet. Moreover, straightforward strategies exist to humanize them, that is to mutate them to their human heavy–chain variable domain equivalent (50). Caplacizumab, a nanobody directed against von Willebrand factor to treat thrombotic thrombocytopenic purpura, was the first clinically approved molecule in Europe and the USA (51).

Since patient-specific autologous therapies such as CAR T cells are advancing, new tools will be required. Tailor-made SyCyRs either supporting or repressing the activity of CAR T cells might help support CAR T-cell–like therapies.

## Experimental procedures

### Generation of anti-idiotypic VHHs

An approximately five-year-old lama (*Lama glama*) was immunized with Palivizumab at preclinics GmbH. All experimental procedures and animal care were in accordance with local animal welfare protection laws and regulations. In brief, for each immunization 300 μg of Palivizumab diluted in a volume of 1 ml PBS were emulsified with either 1 ml Complete Freund’s Adjuvant (first immunization) or Incomplete Freund’s Adjuvant (subsequent immunizations). Injections were administered subcutaneously at three sites. A total of four immunizations were performed over the course of 56 days (0, 28, 42, and 56). On day 60, blood (100 ml) was collected and total RNA was extracted. After the study, camelids remained alive. mRNA, isolated from peripheral B cells of the immunized Lama was transcribed to cDNA utilizing SuperScript III Reverse Transcriptase (Invitrogen) following the manufacturer’s instructions as described previously (52, 53). For PCR amplification of llama VHHs, heavy chain genes were amplified using forward primer CALL001 (5′- GTCCTGGCTGCTCTTCTACAAGG) hybridizing in the region encoding to the leader signal of camelid VH/VHH genes and reverse primer CALL002 (5′ GGTACGTGCTGTTGAACTGTTCC) in the region encoding the CH2 domain. Amplicon DNA was separated by agarose gel electrophoresis and an 800-bp band corresponding to VHH genes was excised, purified using the Promega Wizard SV Gel and PCR Clean-Up System (A9281) and used for a second PCR with primer pair pCT-VHH-Up (5′ GGTGGTGGTGGTTCTGGTGGTGGTGGTTCTGAACAAAAACTCATCTCAGAAGAGGATCTCGGCGGAGGGGGTTCAGATGTGCAGCTGCAGGAGTCTGGRGGAGG) and pCT-VHH-Lo (5′ TACACTGTTGTTATCAGATCTCGACTATTATGAGGAGACGGTGA CCTGGGT). The resulting DNA was introduced into yeast strain EBY100 *via* gap repair of NheI/BamHI linearized pCT vector as described (54). Yeast cells were incubated with Palivizumab for flow cytometry sorting (Fig. S1*A*). To exclude unspecific IgG binders, yeast cells were preincubated with 1 mg/ml Gamunex 10%, a human IgG mixture, followed by incubation with fluorescently labeled anti-human-Fc-phycoerythrin conjugate. These prestained cells were incubated with Palivizumab (60 nM), followed by an anti Fab (κ-chain)-APC conjugate. Cells carrying only the APC fluorescence (Palivizumab-specific) were selected (Fig. S1*B*). After two rounds of sorting, clones were sequenced.

### Cloning of synthetic cytokine ligands and receptors

The cDNAs encoding for the scFv variant of Palivizumab was synthesized by Biocat. The amino acid sequence of Palivizumab was taken from patent US-6955717-B2. The variable heavy and variable light chain sequences were reverse translated into *Homo sapiens*–codon optimized P^scFv^ sequences using EMBOSS Backtranseq V6.6.0. The variable heavy and light chains were separated by a flexible (GGGGS)_4_ linker. The P^scFv^ variants were subcloned into the pcDNA3.1-Fc vector coding for an N-terminal signal peptide and myc tag (EQKLISEEDL) and a C-terminal tobacco etch virus-protease followed by a human IgG1-Fc tag, resulting in expression plasmids pcDNA3.1-P^scFv^LHFc and pcDNA3.1-P^scFv^HLFc. In the tetrameric 2× P^scFv^LHFc, a linker peptide (GGGS) was introduced between the first and second P^scFv^LH. The pcDNA3.1 expression vectors coding for AIP^VHH^ variants contained an N-terminal signal peptide and hemagglutinin-tag, followed by AIP1-4^VHH^ and C-terminal Twin-Strep-tag (WSHPQFEK). AIP^VHH^ were amplified by PCR (Primer forward: 5′-ACTGAGTCCTTAAGGATG TGCAGCTGCAGGAG-3´; Primer reverse: 5′-ATGCGTATGCGGCCGCTGAGGAG ACGGTGACCTG-3′) and cloned *via* AflII and NotI into pcDNA3.1. The resulting plasmids were called pcDNA3.1-AIP1-4^VHH^. pcDNA3.1 expression plasmids for synthetic cytokine receptors were generated by fusion of coding sequence for the IL-11R signal peptide (Q14626, aa 1–22), a myc tag followed by the anti-idiotypic single-domain nanobody (AIP^VHH^1-4), residues of the extracellular domain (ECD), the transmembrane domain (TMD), and intracellular domain (ICD) of the cytokine receptors gp130 and Fas. The amino acids E605 to Q918 from human gp130 (P40189) were used, representing 15 amino acids of the ECD, the TMD, and the ICD. The amino acids C165 to V335 from the human Fas (P25445) were included, representing nine amino acids of the ECD, the TMD, and ICD. AIP1-4^VHH^ were amplified by PCR (Primer forward: 5′-AGTT ACGAGGATCCGATGTGCAGCTGCAGGAG-3´; Primer reverse: 5′-TAGTACGTGAATT CTGAGGAGACGGTGACCTG-3′) and cloned *via* BamHI and EcoRI into pcDNA3.1-SyCyR-gp130, resulting in pcDNA3.1-AIP1-4^VHH^gp130 (6). Subsequently, gp130 cDNA was replaced by Fas cDNA using EcoRI and NotI, resulting in pcDNA3.1-AIP1-4^VHH^Fas. For retroviral transduction of Ba/F3-gp130 cells with the cDNAs coding for the synthetic receptors, the cDNAs were subcloned into pMOWS-puro (7).

### Cells and reagents

The generation of Ba/F3-gp130 cells was described elsewhere (55). The packaging cell line Phoenix-Eco was received from Ursula Klingmüller (DKFZ, Heidelberg, Germany). Cell lines were grown in Dulbecco's modified Eagle's medium high glucose culture medium (GIBCO, Life Technologies) supplemented with 10% fetal bovine serum (GIBCO, Life Technologies), 60 mg/l penicillin, and 100 mg/l streptomycin (Genaxxon bioscience GmbH) at 37 °C with 5% CO_2_. Proliferation of Ba/F3-gp130 cells was maintained in the presence of 0.2% (10 ng/ml) human HIL-6 (24). Expi293F cells (Thermo Fisher Scientific) were cultured in Expi293 expression medium without antibiotics until they reached a density of 3 to 5 × 10^6^ c/ml in a 37 °C incubator with 8% CO_2_ on an orbital shaker at 125 rpm. Phospho-STAT3 (Tyr705) (D3A7; catalog #9145; 1:1000), STAT3 (124H6; catalog #9139; 1:1000), and myc (71D10; cat. #2278) antibodies were obtained from Cell Signaling Technology. StrepMAP-Classic-horseradish peroxidase (HRP) (1:20,000, cat. #2-1509-001) was obtained from IBA GmbH. Rabbit anti-human IgG Fc (#31423) and peroxidase-conjugated secondary mAbs (#31432, #31462) were obtained from Pierce (Thermo Fisher Scientific). Alexa Fluor 488–conjugated Fab goat anti-rabbit IgG (1:500, cat. #4412) was obtained from Cell Signaling Technology. Palivizumab antibody was from Synagis. The cross-linking goat anti-human IgG antibody was obtained from BIOZOL Diagnostics (cat. # SBA-2048-01).

### Transfection of cells

Ba/F3-gp130 cells were retrovirally transduced with the pMOWS expression plasmids coding for AIP1-4^VHH^gp130 and AIP1-3^VHH^Fas variants as described in (7). Transduced cells were grown in Dulbecco's modified Eagle's medium as described above supplemented with 10 ng/ml HIL-6. Selection of transduced Ba/F3-gp130 cells was performed with puromycin (1.5 μg/ml) (Carl Roth) for at least 2 weeks. Afterward, the generated Ba/F3-gp130 cell lines were analyzed for synthetic receptor cell surface expression *via* flow cytometry.

### Mammalian expression and purification of recombinant proteins

pcDNA3.1 encoding P^scFv^Fc variants and AIP^VHH^-Twin-Strep were transfected into Expi293F cells using ExpiFectamine. Reaching 4.5 to 5.5 × 10^6^ c/ml, the cells were diluted to a final density of 3 × 10^6^ c/ml in 30 ml Expi293 expression medium. Thirty micrograms of the plasmid expression vectors were used for transfection according to the manufacturer’s instructions. After 6 days, the culture was harvested by centrifugation at 450*g* at 4 °C for 5 min, followed by centrifugation of the resulting supernatant at 4000*g* at 4 °C for 20 min. The supernatant of the second centrifugation step was filtered (0.45 μm, Carl Roth cat. #P667.1) and purified by affinity chromatography. Recombinant proteins containing a Fc-tag were purified using Protein A resin (1 ml, HiTrap MabSelect PrismA) at a flow rate of 1 ml/min. The column was washed with 30 column volumes of PBS. Proteins were eluted at pH 3.2 to 3.5 using a 50 mM citric acid buffer. Fractions containing the protein peak were pooled, and the pH was adjusted to pH 7 with 1 M Tris, pH 11. Recombinant proteins containing a C-terminal Twin-Strep-tag were purified using Strep-Tactin resin (IBA cat. #2-5025-001) according to the manufacturer’s instructions. All purified proteins were rebuffered to PBS using illustra NAP-25 columns (GE Healthcare Life Sciences). Protein concentrations were determined by measuring absorbance at 280 nm, and samples were flash-frozen in liquid nitrogen. Protein quality was assessed by SDS-PAGE, Coomassie staining, and functional testing.

### Surface plasmon resonance

For surface plasmon resonance experiments, the Biacore X100 instrument (Cytiva Life Sciences) and Protein A sensor chip (Cytiva Life Sciences, #29127558) were used. Palivizumab and P^scFv^Fc variants were captured to a single flow cell at a level of about 1000 or 500 response units per cycle, respectively. Three samples containing only running buffer were injected over both ligand and reference flow cell, followed by AIP1-4^VHH^ serially diluted from 102.4 to 0.1 nM, with an independent final replicate with 6.4 nM. AIP1-4^VHH^ were injected at a flow rate of 30 μl/min for 120 s, and the dissociation was measured for 500 s. Experiments were carried out at 25 °C in PBS pH 7.4, composed of 137 mM NaCl, 2.7 mM KCl, 12 mM HPO_4_^2−^/H_2_PO_4_^−^, and 0.05% (v/v) surfactant P20 (GE Healthcare). The resulting data were reference subtracted and fit to a 1:1 binding model using the Biacore X100 Evaluation software V 2.0.1 (https://cdn.cytivalifesciences.com/api/public/content/digi-48525-pdf).

### SEC-SAXS measurement

We collect the size exclusion chromatography (SEC)-SAXS data on the P12 beamline (PETRA III, DESY Hamburg (56)). The sample to detector distance of the P12 beamline for was 3 m, results in an achievable q-range of 0.03 to 7 nm^−1^. The measurements were performed at 10 °C with a protein concentration of 10 mg/ml for apo Palivizumab and Palivizumab 5.5 mg/ml (37.5 μM) with 1.5 mg/ml (74 μM) AIP1^VHH^ for the complex. The SEC-SAXS runs were performed on a Superdex200 increase 10/300 GL column (100 μl inject, buffer: PBS [137 mM NaCl, 2.7 mM KCl, 12 mM HPO_4_^2−^/H_2_PO_4_^−^, pH 7.4]) with a flowrate of 0.5 ml/min. We collected 3000 frames for each protein sample with an exposer time of 0.995 s/frame. Apo AIP1^VHH^ was measured in batch mode with a concentration of 0.21 mg/ml and an exposer time of 0.095 s/frame (40 frames). Data were scaled to absolute intensity against water. All used programs for data processing were part of the ATSAS software package (Version 3.0.5; https://www.embl-hamburg.de/biosaxs/manuals/install.html) (57). Primary data reduction was performed with the programs CHROMIXS (58) and PRIMUS (59). With the Guinier approximation (60), we determine the forward scattering I(0) and the *R*_*g*_. The program GNOM (61) was used to estimate the maximum particle dimension (*D*_*max*_) with the pair-distribution function *p(r)*.

The model of the Palivizumab IgG was generated out of the available structures. For the FC part, we used a nearly identical IgG (pdb code: 1IGY) as template for the Fc part alignment and for the glycosylation’s. With AlphaFold2 (62, 63) we created the Fc part based on the original sequence of the Palivizumab IgG and realign the two protomers to the 1IGY FC part. We used the glycans from the 1IGY Fc part as template and kept them in the final model. The structure of the Fab part of the Palivizumab IgG is available (pdb code: 2HWZ), and we used this as template for an AlphaFold2 model to fill some gaps in the original structure. The resulting models of the Fc and Fab parts were then used as rigid bodies for the CORAL (63, 64) modeling to obtain representative conformations. The linker regions between the Fc and the Fab part were used as flexible part for the modeling. This is a similar approach as previously described in (65, 66). The nanobody AIP1^VHH^ model was created with AlphaFold2. Only the nanobody core domain was used as a rigid body and the flexible N- and C-terminal parts were remodeled with CORAL (63, 64). The rigid body Fc and Fab position results from apo Palivizumab CORAL model were used as a starting template for the complex docking with AIP1^VHH^. For the AIP1^VHH^ nanobody, we used the core domain as a rigid body and the N- and C-terminal parts as flexible dummies in CORAL (63, 64). After several iterations, we found the docking position of the AIP1^VHH^ nanobody in the Fab part of the Palivizumab. In the final step, we grouped this found position of the AIP1^VHH^ nanobody core domain symmetry equivalent to the Palivizumab Fab domains and did the remodeling with respect to the flexible parts in CORAL again (63, 64).

### Anti-idiotypic (competitive) ELISA

For anti-idiotypic detection, manufacturer’s instructions were followed. Therefore, Palivizumab was coated overnight at 4 °C with a serial dilution starting from 2 to 0.1 μg/ml in 100 μl PBST (0.05% Tween-20). After washing five times with 250 μl PBST, 300 μl 1% bovine serum albumin (BSA) in PBS served as blocking solution for 1 h at room temperature (RT). After removal of blocking solution and washing five times, 100 μl of HRP-conjugated anti-idiotypic detection antibody HCA262P was added at 0.2 μg/ml in 1% BSA PBS for 1 h at RT. For detection plate was washed ten times, 100 μl of detection solution per well was added (Roche Diagnostics GmbH, Mannheim; #35930600) and stopped after 30 min incubation at RT by adding 100 μl stop solution (1.8 M H_2_SO_4_). Infinite M200 PRO plate reader recorded the absorbance at 450 nm with correction at 570 nm. For the refined anti-idiotypic competitive detection, the procedure of commercial anti-idiotypic ELISA was complemented by the following step: the soluble AIP1-4^VHH^ was added at a concentration from 100 to 0.1 nM to the 100 μl containing HRP-conjugated anti-idiotypic detection antibody HCA262P at 0.2 μg/ml.

### Cell surface detection of AIP1-4^VHH^gp130 and AIP1-3^VHH^Fas *via* flow cytometry

5 × 10^5^ Ba/F3-gp130 cells and variants thereof were washed in flow cytometry buffer (PBS, 1% BSA) and then incubated in 50 μl of flow cytometry buffer containing primary myc antibody (1:100). After incubation for 1 h at RT, cells were washed and resuspended in 50 μl of flow cytometry buffer containing secondary antibody (Alexa Fluor 488–conjugated Fab anti-rabbit IgG 1:500) and incubated for 1 h at RT. Cells were washed and resuspended in 500 μl of flow cytometry buffer and analyzed by flow cytometry (BD FACSCanto II flow cytometer using the FACSDiva software, BD Biosciences; https://www.bdbiosciences.com/en-us/products/software/instrument-software/bd-facsdiva-software). Data analysis was conducted using FlowJo Version 10 (Tree Star Inc; https://www.flowjo.com/solutions/flowjo/downloads/previous-versions).

### Proliferation assays

Ba/F3-gp130 cells were washed and 1 × 10^4^ cells were cultured for 3 days in a final volume of 100 μl in the presence of (synthetic) cytokines and antibodies. The CellTiter-Blue Reagent was used to determine cellular viability by recording the fluorescence (excitation 560 nm, emission 590 nm) using an Infinite M200 PRO plate reader (Tecan) immediately after adding 20 μl of reagent per well (time point 0) and up to 120 min thereafter. All conditions were measured in triplicate per experiment. Fluorescence values were normalized by subtraction of time point 0 values. All experiments were performed at least three times, and one representative experiment was selected.

### Stimulation of cells and lysate preparation

10^6^ Ba/F3-gp130 cells and variants thereof/ml were washed three times with PBS and starved in serum-free medium for at least 3 h. Subsequently, cells were stimulated with the indicated (synthetic) cytokines and antibodies for 90 min (or as indicated), harvested by centrifugation at 4 °C for 5 min at 450*g*, frozen, and lysed. Cells were lysed for 2 h with buffer containing 10 mM Tris–HCl, pH 7.8, 150 mM NaCl, 0.5 mM EDTA, 0.5% Nonidet P-40, 1 mM sodium vanadate, 10 mM MgCl_2_, and one complete EDTA-free protease inhibitor mixture tablet (Roche Diagnostics). For JAK inhibition, cells were pretreated for 1 h with 10 μM Pyridone 6 P6 (JAK inhibitor; Sigma-Aldrich #420097). Protein concentration of cell lysates was determined by the bicinchoninic acid Protein Assay (Pierce, Thermo Fisher Scientific).

### Western blotting

Proteins were separated by SDS-PAGE and transferred to polyvinylidene difluoride or nitrocellulose membranes for 60 min (20 V, 1 A). Membranes were blocked and probed with the indicated primary antibodies. After washing, membranes were incubated with secondary peroxidase–conjugated antibodies (1:2500) or fluorescence-labeled secondary antibodies (1:10,000). The Immobilon Western Reagents (Millipore Corporation) and the ChemoCam Imager (INTAS Science Imaging Instruments GmbH) or the Odyssey Fc Imaging System (LI-CORE Biosciences) were used for signal detection.

### AnnexinV/7-aminoactinomycin D staining

Ba/F3/gp130 cell lines were washed three times with PBS. 1.25 × 10^5^ cells were used per well and incubated with the indicated cytokines for 24 h or 48 h. For the ethanol condition, cells were only incubated with HIL6 (10 ng/ml). Ethanol treatment replaced the last washing step before the measurement. Cells were washed twice with ice-cold PBS and if indicated with 70% ethanol. Cells were resuspended in 300 μl Annexin V binding buffer (BD Bioscience) with 0.5 μl Annexin V-PE (ImmunoTools) and incubated for 15 min in the dark at RT. One microliter 7-aminoactinomycin D (R&D Systems) was added before analysis was carried out by flow cytometry recording 20,000 events.

### Fluorimetric caspase 3/7 assay

Ba/F3-gp130 cells were washed three times with PBS. 1.25 × 10^5^ cells were incubated with the indicated cytokines for 6 h in a 96-well plate in a volume of 100 μl. Subsequently, induction of apoptosis was determined using Amplite Fluorimetric Caspase 3/7 Assay kit (AAT Bioquest, Inc) according to manufacturer´s recommendations. In brief, 100 μl of the caspase-3/7 working solution was added to the cells and incubated for 2 h at RT. After centrifugation at 450*g* for 1 min, the fluorescence (excitation 350 nm, emission 450 nm) using an Infinite M200 PRO plate reader (Tecan) was determined.

### Statistical analyses

For proliferation assays, a representative experiment of n ≥3 assays with comparable results is displayed. EC_50_ or IC_50_ values were determined using a nonlinear regression analysis with variable slope calculation in GraphPad Prism 8.0 (version 8.0.2 for Windows, GraphPad Software, www.graphpad.com) from three individual experiments. The data are presented as means ± SD. For multiple comparisons, two-way ANOVA including Bonferroni as statistical hypothesis test was used (GraphPad Prism 8.0.2, GraphPad Software Inc). Statistical significance was set at the level of *p* <0.05 (∗*p* < 0.05; ∗∗*p* < 0.01; and ∗∗∗*p* < 0.001).

## Data availability

We will upload the SAXS data to the Small Angle Scattering Biological Data Bank (67).

## Supporting information

This article contains supporting information.

## Conflict of interest

The authors declare that they have no conflicts of interest with the contents of this article.
