# Supplementary material for: Respiratory syncytial virus–approved mAb Palivizumab as ligand for anti-idiotype nanobody-based synthetic cytokine receptors
Source: J Biol Chem. 2023 Sep 19;299(11):105270. doi: 10.1016/j.jbc.2023.105270 (PMC10630626; doi:10.1016/j.jbc.2023.105270)

Supplemental Figure 1

A

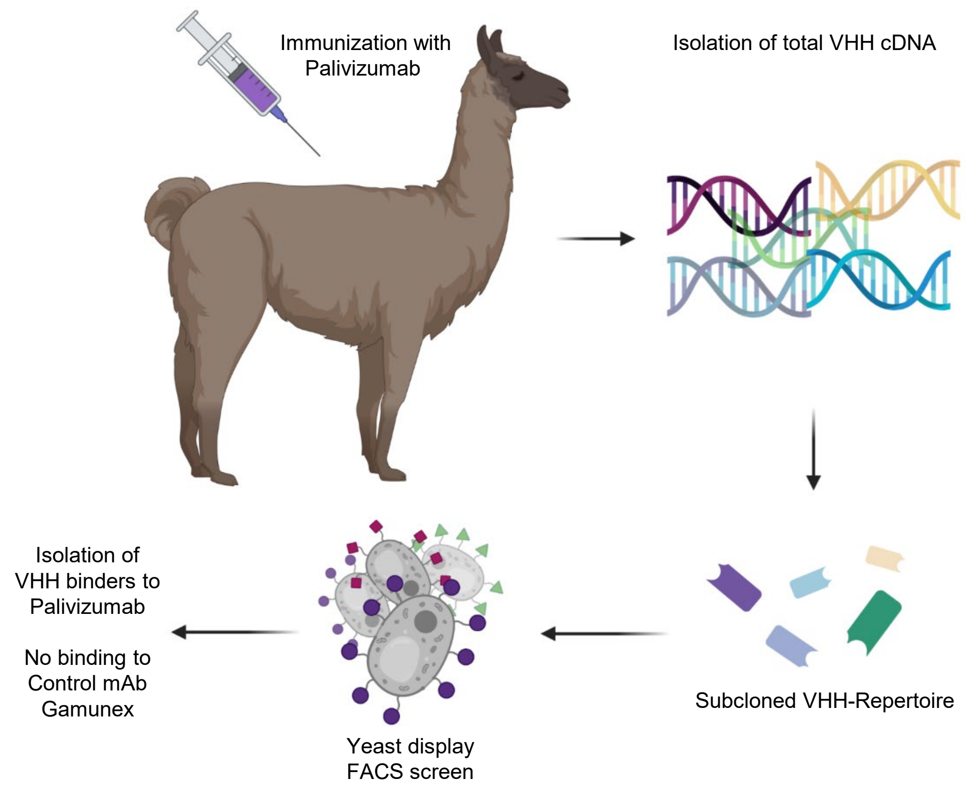

B

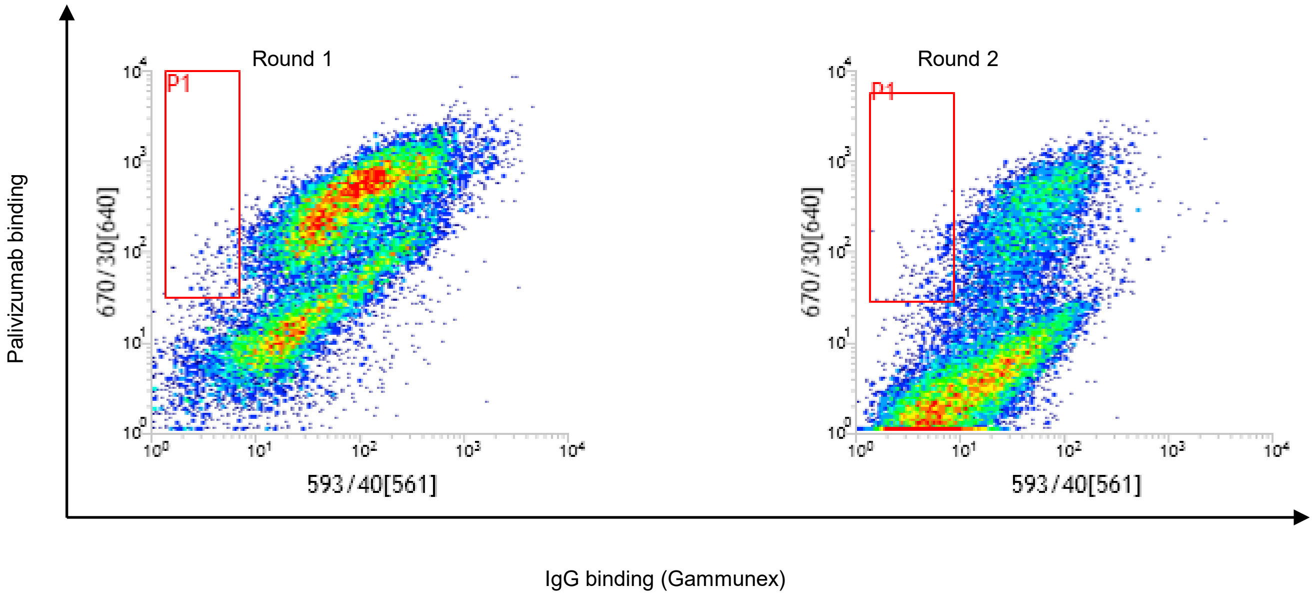

Supplemental Figure 2

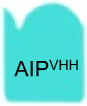


```
                                                  CDR1                                CDR2
                10         20         30         40         50         60
AIP1VHH  DVQLQESGGG LVQAGGSLRL SCVASGLTF- RYDMGWFRQA PGKEREFVAQ ITW-RGGSAT
AIP2VHH  DVQLQESGGG LVQAGGSLRL SCAASESLFR LNAMGWYRQA PGKQRELVAG IT---TSGDA
AIP3VHH  DVQLQESGGG LVQPGGSLRL SCVASGRTWS IYAMGWFRQA PGKEREFVTA ISISRSESVT
AIP4VHH  DVQLQESGGG LVQAGGSLRL SCVASGRAFS RYDMGWFRQA PGKEREFVAQ ISW-RGGSAT

         CDR2                                              CDR3
                  70         80         90        100        110        120
AIP1VHH   YYADSVKGRF TIARDIAKNA VYLQMNSLKP EDTAVYYCAA AYGSAGYRPD EYDSWGQGTQ VTVSS
AIP2VHH   VYADSVKGRF TISRDSAKNT VYLQTNNLKP EDTAVYYCNV RPRAGTWENA RIGVWGQGTQ VTVSS
AIP3VHH   HYAESVTGRF TISRDNAKNT LHLQMNSLKP EDTAVYFCAA DRRSID-PHH TIDYWGQGTQ VTVSS
AIP4VHH   SYADTVKGRF TIARDNAKNT VYLQMNSLKP EDTAVYYCNA --------RTP TLSSWGQGTQ VTVSS
```

Supplemental Figure 3

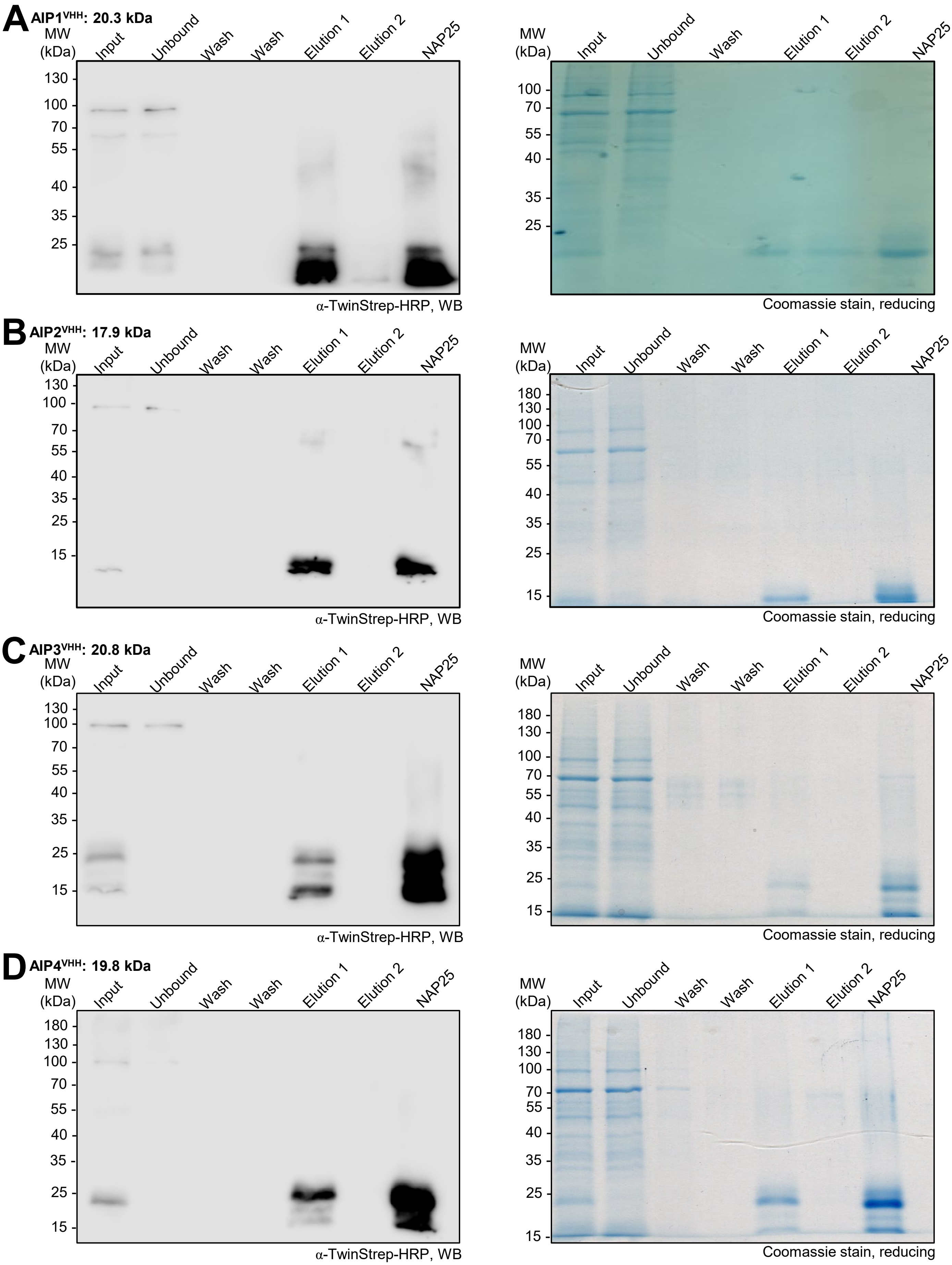

Supplemental Figure 4

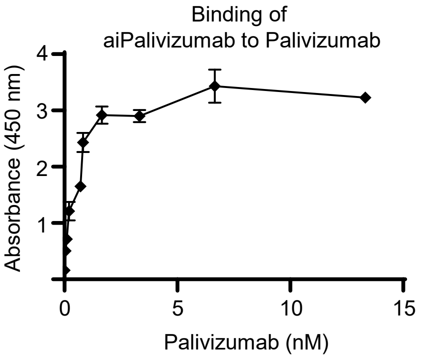

Supplemental Figure 5

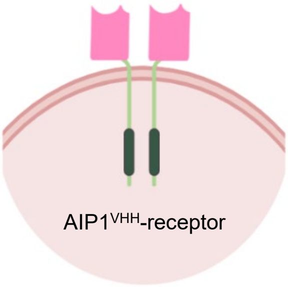

AIP1^{VHH}-gp130

MSSSCSGLSRVLVAVATALVSASSEQKLISEEDLGSDVQLQESGGGLVQAGGSLRLSCVASGLTFRYDMGWFRQAPGKE
REFVAQITWRGGSATYYADSVKGRFTIARDIAKNAVYLQMNSLKPEDTAVYYCAAAYGSAGYRPDEYDSWGQGTQVTVS
SEFTFTTPKFAQGEIEAIVVPVCLAFLLTTLLGVLFCFNKRDLIKKHIWPNVPDPSKSHIAQWSPHTPPRHNFNSKDQM
YSDGNFTDVSVVEIEANDKKPFPEDLKSLDLFKKEKINTEGHSSGIGGSSCMSSSRPSISSSDENESSQNTSSTVQYST
VVHSGYRHQVPSVQVFSRSEATQPLLDSEERPEDLQLVDHVDGGDGILPRQQYFKQNCSQHESSPDISHFERSKQVSSV
NEEDFVRLKQQISDHISQSCGSGQMKMFQEVSAADAFGPGTEGQVERFETVGMEAATDEGMPKSYLPQTVRQGGYMPQA
AARV

Signal peptide – Myc – AIP1^{VHH} – gp130

AIP1^{VHH}-Fas

MSSSCSGLSRVLVAVATALVSASSEQKLISEEDLGSDVQLQESGGGLVQAGGSLRLSCVASGLTFRYDMGWFRQAPGKE
REFVAQITWRGGSATYYADSVKGRFTIARDIAKNAVYLQMNSLKPEDTAVYYCAAAYGSAGYRPDEYDSWGQGTQVTVS
SEFMCKEEGSRSNLGWLCLLLLPIPLIVWVKRKEVQKTCRKHRKENQGSHESPTLNPETVAINLSDVDLSKYITTIAGV
MTLSQVKGFVRKNGVNEAKIDEIKNDNVQDTAEQKVQLLRNWHQLHGKKEAYDTLIKDLKKANLCTLAEKIQTIILKDI
TSDSENSNFRNEIQSLV

Signal peptide – Myc – AIP1^{VHH} – Fas

Supplemental Figure 6

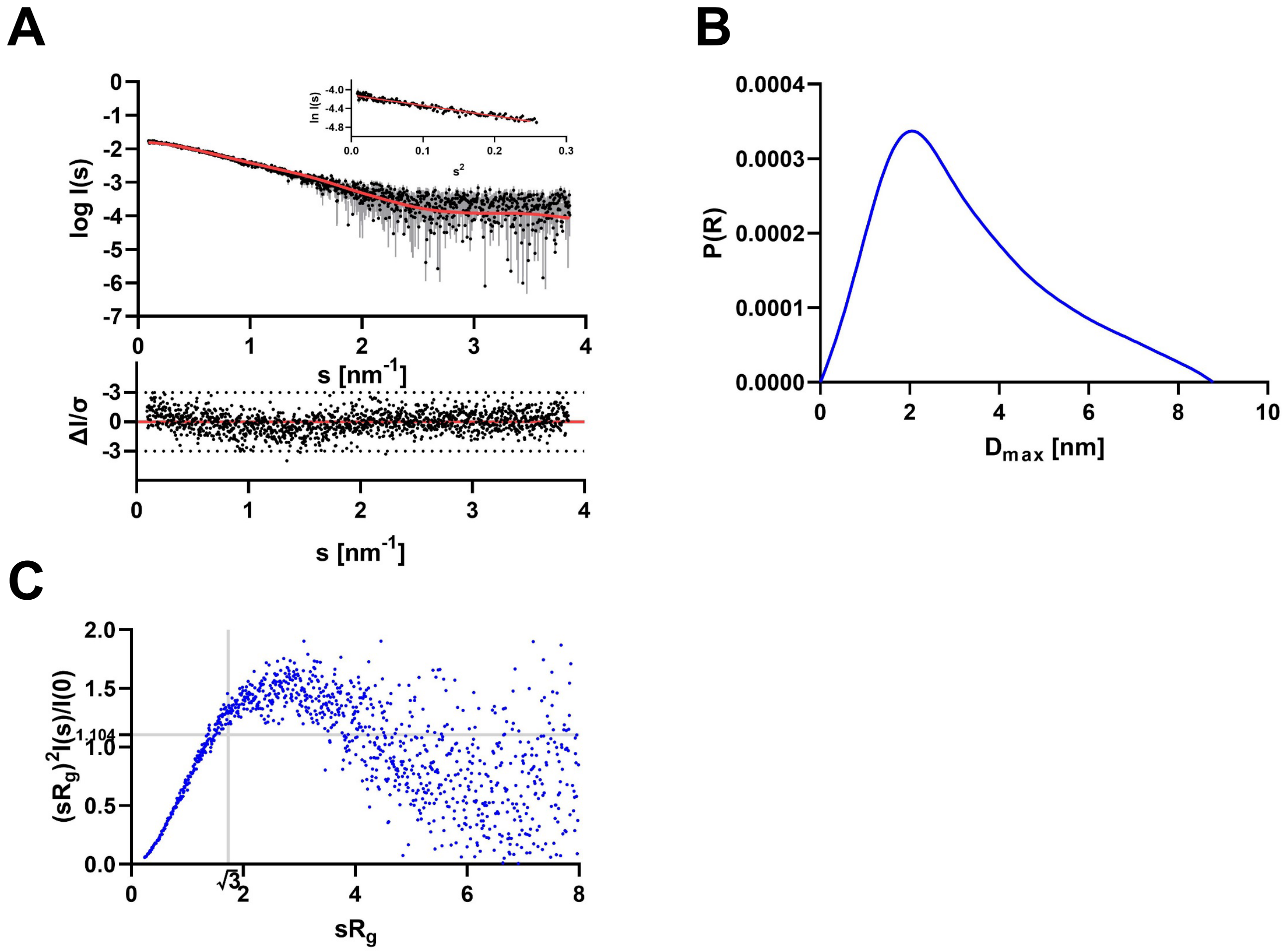

Supplemental Figure 7

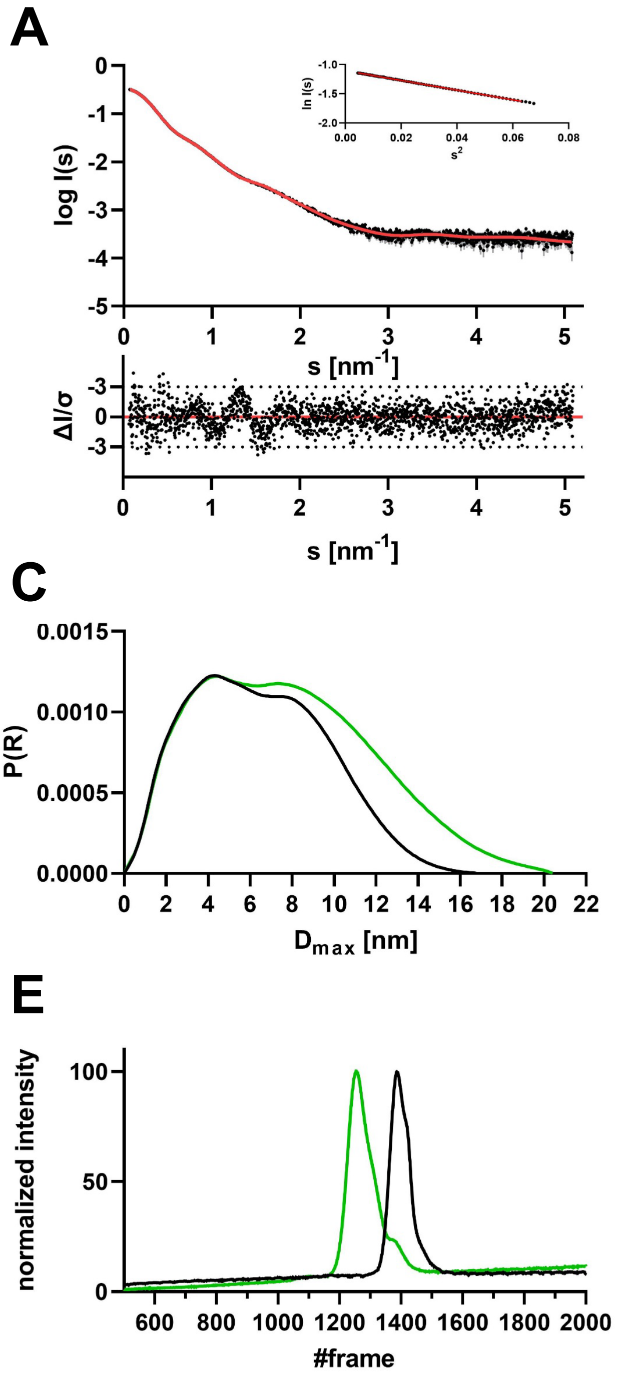

Supplemental Figure 8

A

Palivizumab

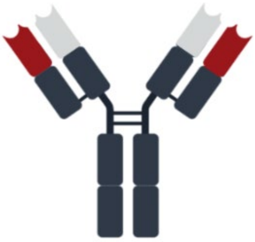

150 kDa
Epitope distance: 146Å

P^{scFv}LHFc

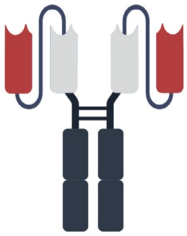

107 kDa
Epitope distance: 90Å
LH-orientation
Linker: 23 aa

P^{scFv}HLFc

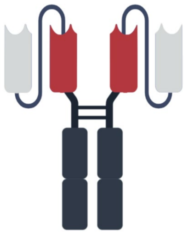

105 kDa
Epitope distance: 114Å
HL-orientation
Linker: 15 aa

B

P^{scFv}LHFc

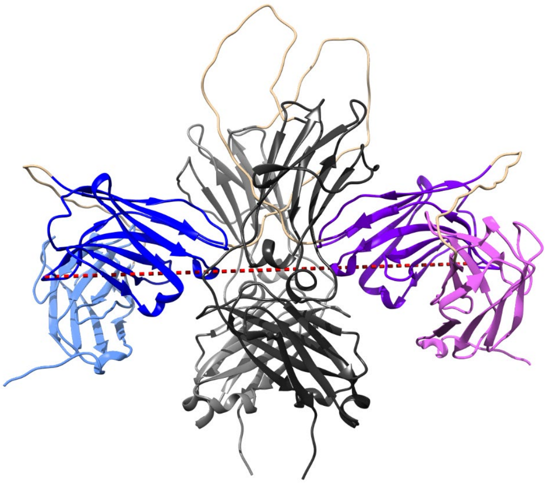

107 kDa
Paratope
Paratope: 90Å
LH-orientation
Linker: 23 aa

P^{scFv}HLFc

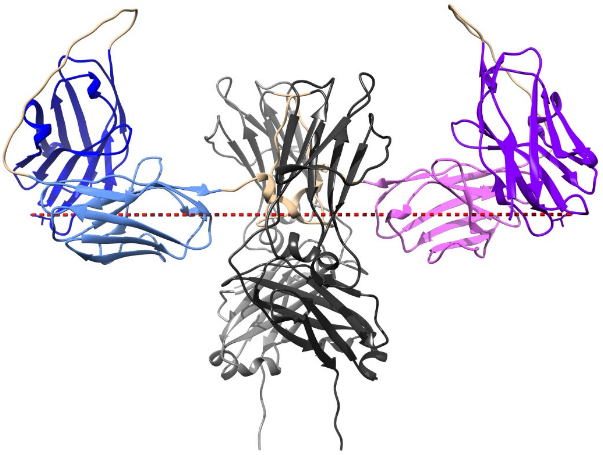

105 kDa
Paratope
Paratope: 114Å
HL-orientation
Linker: 15 aa

Supplemental Figure 9

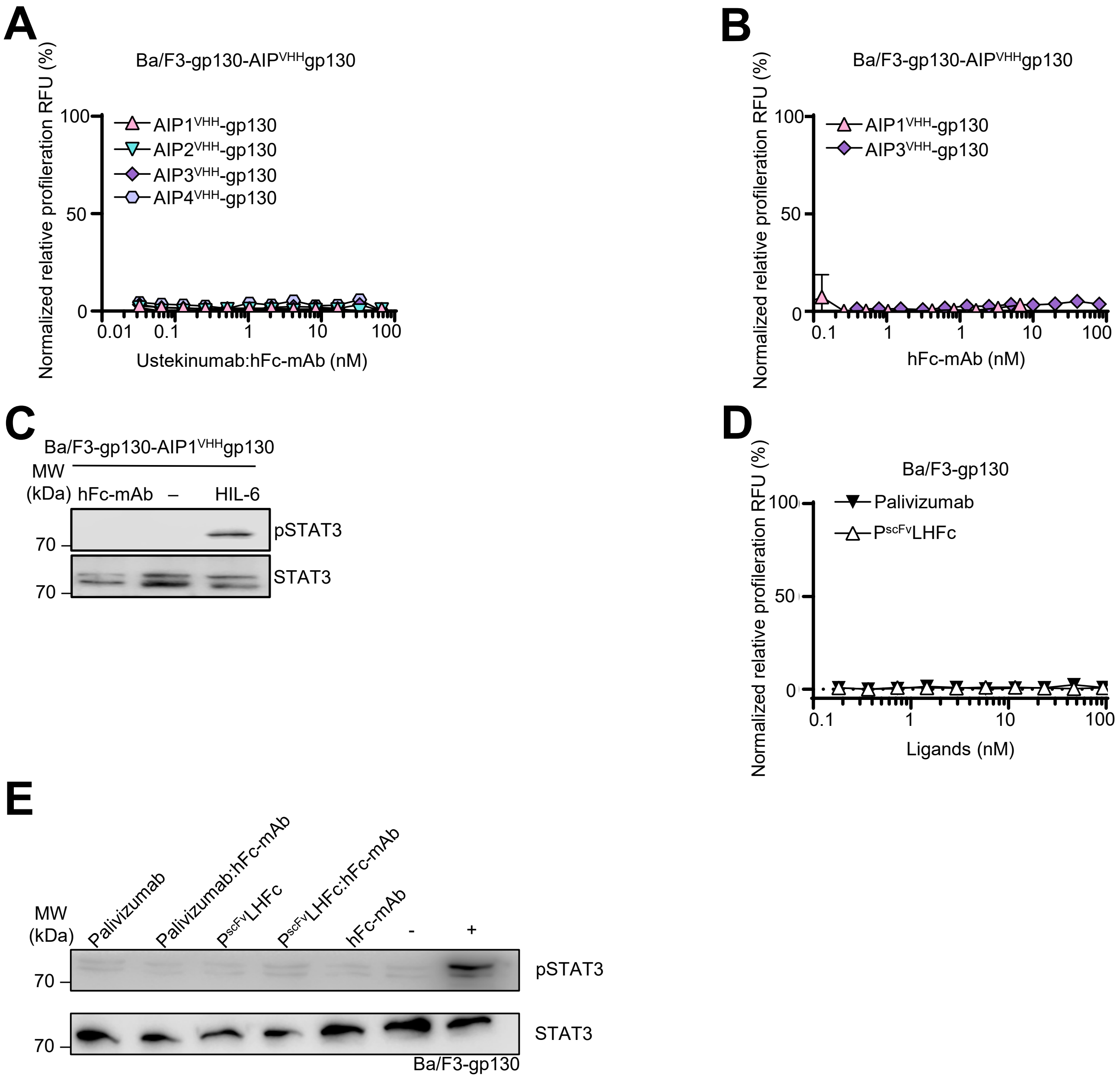

Supplemental Figure 10

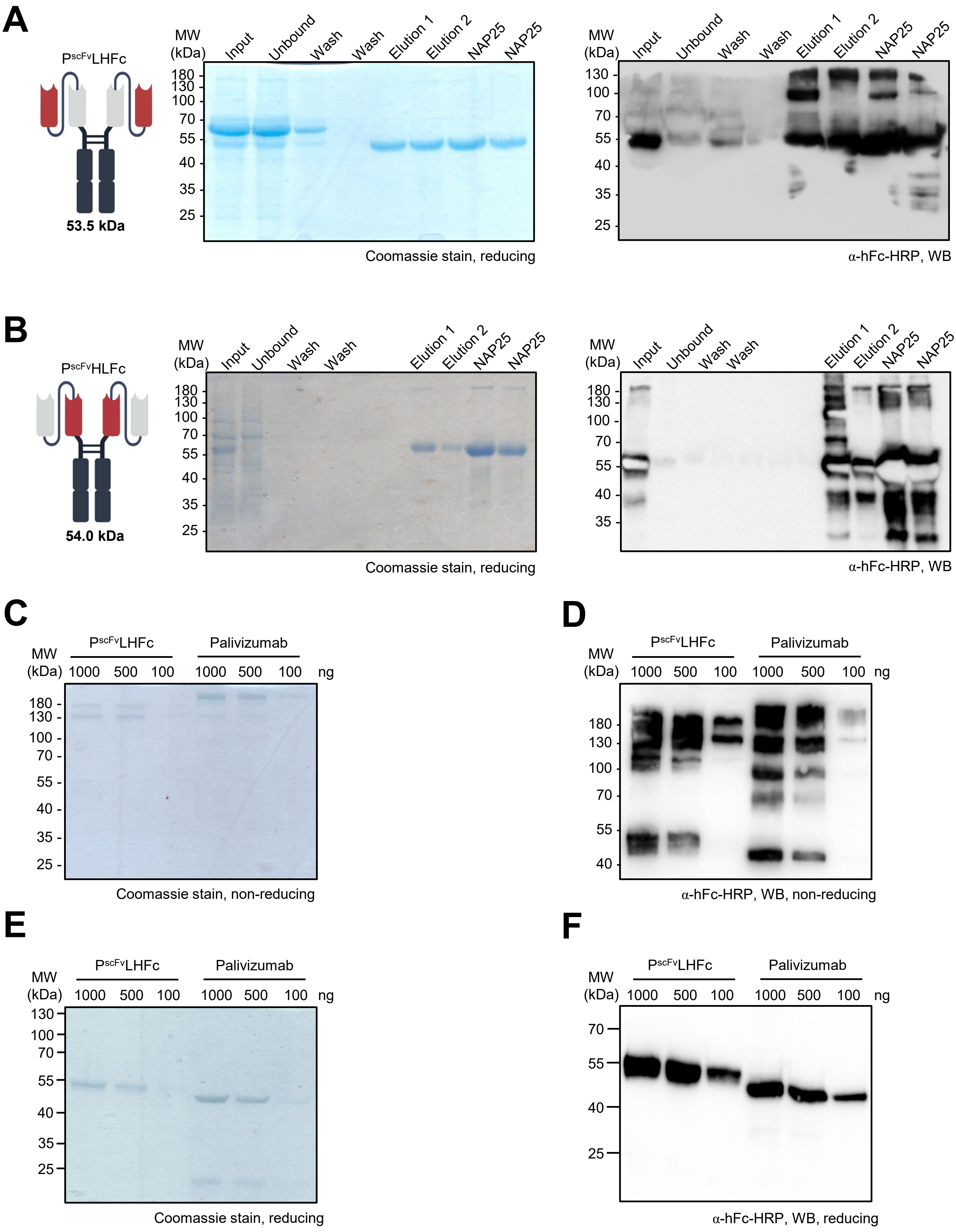

Supplemental Figure 11

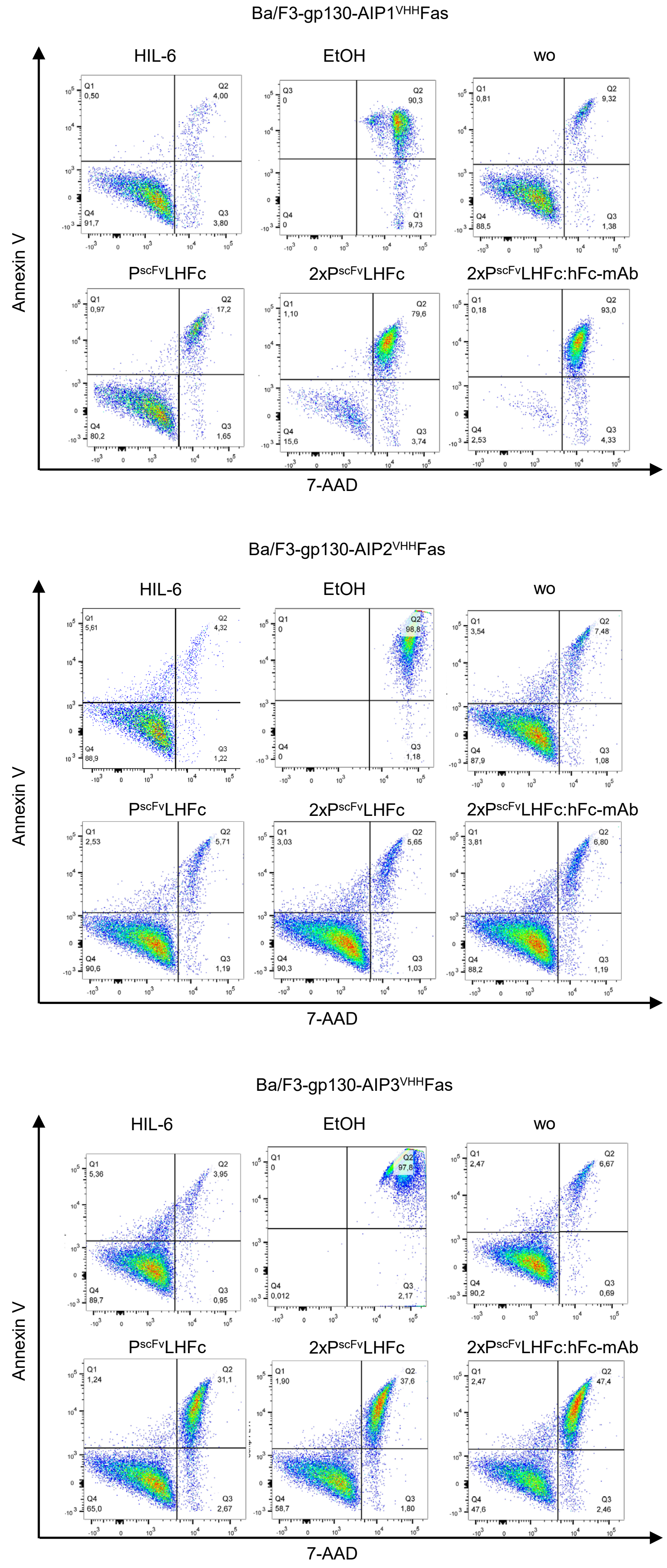

Supplement: Supporting Figures S1–S11 [file mmc1.pdf]
