# Supplementary material for: Respiratory syncytial virus–approved mAb Palivizumab as ligand for anti-idiotype nanobody-based synthetic cytokine receptors
Source: J Biol Chem. 2023 Sep 19;299(11):105270. doi: 10.1016/j.jbc.2023.105270 (PMC10630626; doi:10.1016/j.jbc.2023.105270)
Supplement: Supporting Table S1 [file mmc2.docx]

**Table S1: Overall SAXS Data**

| Data collection parameters |  | | |
| --- | --- | --- | --- |
| SAXS Device | P12, PETRA III, DESY Hamburg (*56*) | | |
| Detector | PILATUS 6 M (423.6 x 434.6 mm^2^) | | |
| Detector distance (m) | 3.0 | | |
| Beam size | 120 µm x 200 µm | | |
| Wavelength (nm) | 0.124 | | |
| Sample environment | Quartz glass capillary, 1 mm ø | | |
| Absolute scaling method | Comparison with scattering from pure H_2_O | | |
| Normalization | To transmitted intensity by beam-stop counter | | |
| Scattering intensity scale | Absolute scale, cm^-1^ | | |
| *s* range (nm^-1^)^‡^ | 0.03 – 7.0 | | |
| Sample | Palivizumab | Nanobody AIP1^VHH^ | Complex |
| Organism | Commercial (Synagis) | Lama glama | - |
| Mode of measurement | online SEC-SAXS | batch | online SEC-SAXS |
| SEC-Column | Superdex200 increase 10/300 GL | - | Superdex200 increase 10/300 GL |
| Flowrate (ml/min) | 0.5 | - | 0.5 |
| Injection volume (µl) | 100 | 50 | 100 |
| Temperature (°C) | 10 | | |
| Exposure time (# frames) | 0.995 s (3000) | 0.095 s (40) | 0.995 s (3000) |
| # frames used for averaging | 22 | 38 | 15 |
| Protein buffer | PBS (137 mM NaCl, 2.7 mM KCl, 12 mM HPO_4_^2−^/H_2_PO_4_^−^, pH 7.4) | | |
| Protein concentration (mg/ml) | 10 | 0.21 | 5.5 Palivizumab  1.5 Nanobody AIP1^VHH^ |
| Structural parameters |  | | |
| *Guinier Analysis (PRIMUS)* |  | | |
| *I*(0) ± σ (cm^-1^) | 0.33 ± 0.000122 | 0.016 ± 0.0001 | 0.17 ± 0.00017 |
| *R*_g_ ± σ (nm) | 4.99 ± 0.006 | 2.58 ± 0.03 | 5.97 ± 0.01 |
| *s-range* (nm^-1^) | 0.067 – 0.224 | 0.092 – 0.500 | 0.103 – 0.218 |
| *min < sRg < max limit* | 0.33 – 1.30 | 0.24 -1-29 | 0.60 – 1.29 |
| Data point range | 1 - 70 | 1 - 147 | 1 - 43 |
| Linear fit assessment (R^2^) | 0.9998 | 0.9381 | 0.9994 |
| *PDDF/P(r) Analysis (GNOM 5)* |  | | |
| *I*(0) ± σ (cm^-1^) | 0.33 ± 0.00011 | 0.02 ± 0.000087 | 0.17 ± 0.00017 |
| *R*_g_ ± σ (nm) | 5.06 ± 0 0026 | 2.61 ± 0.0188 | 6.17 ± 0.0075 |
| *D*_max_ (nm) | 16.76 | 8.78 | 20.41 |
| Porod volume (nm^3^) | 252.29 | 30.96 | 400.00 |
| *s-range* (nm^-1^) | 0.067 – 5.09 | 0.092 – 3.86 | 0.103 – 4.85 |
| χ2 / CorMap P-value | 1.059 / 0.053 | 0.962 / 0.482 | 1.037 / 0.186 |
| Molecular mass (kDa) |  | | |
| From *I*(0) | n.d. | 20.62 | n.d. |
| From Qp (*18*) | 153.96 | 20.39 | 209.52 |
| From MoW2 (*19*) | 153.40 | 20.01 | 175.65 |
| From Vc (*20*) | 141.34 | 19.04 | 178.24 |
| Bayesian Inference (*21*) | 146.80 | 19.93 | 185.58 |
| From sequence | 144.92 | 20.25 | 185.42- |
| Rigid body modeling |  | | |
| CORAL |  |  |  |
| Symmetry | P1 | P1 | P1 |
| *s-*range for fit (s*_min_* – s*_max_*; nm^-1^) | 0.067 – 5.09 | 0.092 – 3.86 | 0.101 – 5.13 |
| *χ* ^2^, CorMap *P*-value | 1.468 / 0.000007 | 1.108 / 0.00001 | 1.272 / 0.000007 |
| SASBDB accession codes (*64*) | SASDSU6 | SASDSV6 | SASDSW6 |
| Software |  | | |
| ATSAS Software Version (*57*) | 3.0.5 | | |
| Primary data reduction | CHROMIXS (*58*)/ PRIMUS (*59*) | | |
| Data processing | GNOM (*61*) | | |
| *Rigid body* modeling | CORAL (*64*) | | |
| Model visualization | PyMOL (*22*) | | |

‡s = 4πsin(θ)/λ, 2θ – scattering angle, n.d. not determined
